# Disuse‐Induced Muscle Atrophy and Muscle Weakness From Hospitalization to Spaceflight: Exercise Succeeds in Prevention and Treatment—A Meta‐Analysis

**DOI:** 10.1002/jcsm.70259

**Published:** 2026-04-15

**Authors:** Camila S. Padilha, Caique Figueiredo, Rafael Deminice, Alex S. Ribeiro, Robert U. Newton, Nicolas H. Hart, José Cesar Rosa‐Neto, Marília Seelaender, Fábio Santos Lira

**Affiliations:** ^1^ Centre for Healthy Ageing, Biology of Ageing Laboratory, Centenary Institute of Cancer Medicine and Cell Biology Royal Prince Alfred Hospital Sydney NSW Australia; ^2^ Faculty of Medicine and Health, Charles Perkins Centre University of Sydney Sydney NSW Australia; ^3^ School of Sport, Exercise and Rehabilitation Science, Faculty of Health University of Technology Sydney (UTS) Sydney NSW Australia; ^4^ Exercise and Immunometabolism Research Group, Postgraduation Program in Movement Sciences, Department of Physical Education Universidade Estadual Paulista (UNESP), Presidente Prudente São Paulo Brazil; ^5^ Immunometabolism Research Group, Department of Cell Biology and Developmental, Institute of Biomedical Sciences University of São Paulo (USP) São Paulo Brazil; ^6^ Laboratory of Biochemistry Exercise, Department of Physical Education, Faculty of Physical Education and Sport State University of Londrina Londrina Brazil; ^7^ Faculty of Sports Sciences and Physical Education, CIPER University of Coimbra Coimbra Portugal; ^8^ Exercise Medicine Research Institute Edith Cowan University Joondalup WA Australia; ^9^ School of Medical and Health Sciences Edith Cowan University Joondalup WA Australia; ^10^ Human Performance Research Centre, INSIGHT Research Institute, Faculty of Health University of Technology Sydney (UTS) Sydney NSW Australia; ^11^ Cancer Metabolism Research Group, Department of Surgery, LIM26‐HC and C2PO, Faculdade de Medicina, CoMeta‐HU University of São Paulo São Paulo Brazil

**Keywords:** atrophy, functional capacity, immobility, resistive exercise, unloaded muscle

## Abstract

**Background:**

Exercise has been proposed as both a preventive and therapeutic countermeasure; however, its effectiveness across different disuse conditions and timings of implementation remains uncertain.

**Methods:**

This systematic review and meta‐analysis (PROSPERO: CRD42021256599) searched ClinicalTrials.gov, Cochrane Central, PubMed, SPORTDiscus, Web of Science, Scopus, CINAHL and SciELO from inception to May 2021, with an update in March 2025. Randomized and nonrandomized controlled trials examining exercise interventions during or after muscle disuse were included according to the PICOS framework. Random‐effects meta‐analysis evaluated effects on muscle strength, power and mass across hospitalization, bed rest and spaceflight conditions. Effect sizes (ES) are reported as standardized mean differences with 95% confidence intervals (CI).

**Results:**

A total of 1754 participants (66% male, 34% female; mean age 49 ± 22 years) were included. Preventive exercise interventions significantly improved muscle strength and power across disuse models. During hospitalization, exercise significantly increased muscle strength (ES = 0.60, 95% CI [0.42, 0.78]; *p* < 0.0001; *I* ^2^ = 76%). This effect remained significant in multilevel analyses accounting for within‐study dependence (ES = 0.58, 95% CI [0.19, 0.96]; *p* = 0.003). During bed rest, conventional random‐effects analyses indicated large effects (ES = 1.16, 95% CI [0.60, 1.71]; *p* < 0.0001; *I* ^2^ = 55%); however, multilevel models incorporating correlated lower limb strength and power outcomes showed no significant pooled effect (ES = 0.06, 95% CI [−1.31, 1.43]; *p* = 0.93). In contrast, spaceflight studies demonstrated small, nonsignificant effects on muscle strength and power (ES = 0.10, 95% CI [−0.32, 0.51]; *p* = 0.65), and pooled standardized mean change analyses indicated no significant overall change in muscle mass (ES = 0.002, 95% CI [−0.077, 0.080]; *p* = 0.966). When exercise was applied therapeutically after disuse, a trend toward improved muscle strength and power was observed (ES = 0.23, 95% CI [−0.01, 0.47]; *p* = 0.06; *I* ^2^ = 12%), although multilevel models showed no significant effects (ES = 0.30, 95% CI [−0.34, 0.95]; *p* = 0.361). Exercise significantly preserved or increased muscle mass during bed rest (ES = 0.47, 95% CI [0.19, 0.74]; *p* = 0.0009) and spaceflight (ES = 0.27, 95% CI [0.05, 0.48]; *p* = 0.02).

**Conclusion:**

Exercise, particularly resistance training, attenuates muscle strength loss and preserves muscle mass during hospitalization and bed rest, whereas no consistent benefits are observed during spaceflight. Exercise initiated after disuse shows modest potential for restoring muscle function. Current evidence is insufficient to determine whether preventive or therapeutic initiation provides superior outcomes. Further high‐quality randomized controlled trials are required to define the optimal timing, modality and dose of exercise for clinical rehabilitation and aerospace applications.

## 1 Introduction

Prolonged physical inactivity due to acute hospitalization, bed rest or spaceflight missions can lead to muscle atrophy and weakness [1, 2, 3, 4]. Hospitalized older adults typically experience muscle atrophy and weakness, contributing to prolonged recovery time and increased risk of functional decline and frailty [5, 6, 7]. Furthermore, disuse‐induced sarcopenia renders surgical outcomes less successful [8, 9]. Similarly, young adults undergoing extended bed resting periods exhibit rapid loss in muscle function and mass, while astronauts during spaceflight missions present significant musculoskeletal deconditioning despite existing countermeasures [2, 10, 11]. Muscular strength and muscle mass significantly impact the survival rate of individuals [5, 12]. International consensus has established that muscle mass and strength assessments robustly predict outcomes in chronic disease [13]. Given these challenges, identifying effective strategies to mitigate muscle loss and restore function is crucial for improving patient recovery and maintaining astronaut health.

Skeletal muscle mass plays an essential role in numerous vital functions, including locomotion and metabolic homeostasis [14, 15, 16]. Sarcopenia has long been associated with ageing and older people, but the development of sarcopenia is now recognized to begin earlier in life [17]. The updated consensus on sarcopenia of the European Working Group of Sarcopenia in Older People (EWGSOP) requires documentation of low muscle strength and low muscle mass, while physical performance is used to categorize the severity of sarcopenia, which has implications for interventions that prevent or delay the development of neuromuscular disability [1]. People with acute medical illness requiring subsequent hospitalization reportedly experience a rapid decline in muscle strength (~10.9%) and muscle volume (~7.8%) after 5–12 days of in‐hospital care as a result of the inactivity [18, 19, 20]. Similarly, astronauts experience a reduction in quadriceps muscle volume (~15%) and a decline in muscle strength after 28 weeks of a spaceflight mission [10, 21]. It is noteworthy that the deficit in muscle mass and functional capacity may persist for months or years after hospital discharge [22] and in astronauts after long‐duration spaceflight mission [23]. Therefore, strategies pursuing the attenuation of disuse‐induced muscle atrophy are vital, preventing loss of quality of life and diminishing the cost involved in the mitigation of the consequences of inactivity or low gravity.

Regular exercise is an acknowledged low‐cost and side‐effect‐free strategy to improve skeletal muscle health [24, 25]. Exercise, and in particular, resistance exercise, when employed as a preventive strategy, is effective in attenuating muscle function decline in hospitalized individuals. Similarly, astronauts candidates must undergo a programme of physical conditioning before long‐duration spaceflights to maintain physical fitness and facilitate reconditioning upon return to Earth [26]. A combination of resistance and aerobic exercise is the most predominant programme adopted to improve skeletal muscle health [27, 28]. However, it remains unclear if exercise, as a preventive strategy or as a therapeutic strategy for muscle recovery, would be sufficient to counteract muscle function and mass decline in individuals undergoing hospitalization, bed rest or spaceflight. Accordingly, we summarize the evidence on the effects of exercise in preventive or treatment strategies for maintaining neuromuscular strength and muscle mass in disuse‐induced muscle atrophy.

## 2 Methods

### 2.1 Data Sources and Searches Approach

We followed the Preferred Reporting Items for Systematic Reviews and Meta‐Analysis (PRISMA) guidelines [29] (see Data S1) and registered this study in the International Prospective Register of Systematic Reviews (PROSPERO identified: CRD42021256599). All articles were required to be written in English for inclusion. We retrieved articles by title and abstract from the earliest record up to March 2025 on Clinical Trial Register, Cochrane Trial Register, PubMed, SPORT Discus, Web of Science, Scopus, SciELO and Cumulative Index to Nursing Allied Health (CINAHL) by two independent authors (CSP and CF). Within these databases, the following search terms and phrases were combined: (acute hospitalization OR hospitalized individuals OR hospital‐based care OR inpatients OR posthospitalization OR after hospitalization OR outpatient AND bed rest OR bedridden individuals OR limb‐immobilization OR immobilization OR hindlimb OR postbed rest OR post immobilization OR hindlimb rehabilitation AND spaceflight missions OR aerospace flight OR long‐duration space mission OR international space station OR astronauts OR postspaceflight mission OR astronauts rehabilitation OR postaerospace flight AND physical exercise OR aerobic training OR endurance training OR resistance training OR structured exercise training OR combined exercise training OR resistance exercise OR multicomponent exercise OR weight training OR interim resistive exercise device OR resistive device AND muscle mass OR lean body mass OR muscle size OR cross‐sectional area OR muscle strength OR muscular strength OR muscle force OR muscle power OR dynamic muscle strength OR handgrip strength OR muscle contraction OR maximum voluntary contraction AND (‘Random*’ OR ‘control*’ OR ‘clinic*’)). The search strategy and full‐detailed database search is available (see Data S2). Retrieved titles and abstracts were identified by cross‐referencing of selected articles by two independent researchers (CSP and CF) to assess eligibility for meta‐analysis. In case of disagreements, a third researcher (RD) was consulted to evaluate the article.

### 2.2 Eligibility Criteria

Eligibility criteria were determined in accordance with the Population, Intervention, Comparators, Outcome and Study (PICOS design) framework. In detail, we included studies which (P) individuals who were experiencing or recovering from hospitalization, bed rest or spaceflight; (I) structured exercise therapy as a preventive and/or treatment strategy; (C) any control group, usual care or nonstructured exercise; and in the case of spaceflights, an absence of control groups necessitated comparison against baseline values; (O) neuromuscular strength, power and function, and muscle mass parameters; and (S) controlled trial designs (randomized, RCT; and nonrandomized, NRCT). After screening eligible full texts, we excluded articles that engaged children, pregnant women or participants undergoing chronic pharmacological treatment, wrong outcome, incomplete end points, different comparators and studies presenting a high risk of bias (> 4 domains out of 6) (see Data S3). Agreement between researchers for the title and abstract screening was considered very good (kappa = 0.915; < 0.001).

### 2.3 Data Extraction and Quality Assessments

Quality of randomized controlled trials was assessed using the Risk of Bias (RoB) assessment tool of the Cochrane Collaboration [30] and nonrandomized studies was performed using the risk of bias in nonrandomized studies of interventions (ROBINS:I) assessment tool of the Cochrane Collaboration [31]. The quality of selection bias, performance bias, detection bias, attrition bias, reporting bias and other bias were classified as high (‘+’), low (‘−’), or unclear (‘?’) RoB [30]. Quality assessments of two independent researchers (CSP and CF) were compared, and potential disagreements were resolved by discussion (see Data S4). The following information was extracted from each study for analysis: author/year, the number of participants within each group and baseline participant characteristics, intervention details, baseline and postdata from all outcomes. In circumstances where standard deviations (SD) were not available, these values were calculated using traditional statistical methods, assuming a correlation of 0.5 between baseline and postintervention scores within each subject [32], and a validated online platform, WebPlotDigitizer (https://automeris.io/WebPlotDigitizer/) was used to obtain mean and SD values from figures where possible [33, 34]. Similarly, when studies reported the standard error, the values were converted to standard deviation (SD) according to the Hozo's equations [35].

### 2.4 Data Syntheses and Analysis

Meta‐analysis was conducted using Review Manager (RevMan 5.4) to calculate the exercise effect size (ES) in RCTs studies as a preventive and therapeutic strategy on muscular strength and muscle mass during hospitalization in older patients, bed rest in young adults or NRCTs studies in middle‐aged astronauts during spaceflight missions. Studies without a control group (3/6 spaceflight studies) were analysed using prespaceflight and postspaceflight values (as self‐controls), which consider only the intervention group. To account for statistical dependence from multiple effect sizes within studies, analyses were performed using multilevel meta‐analytic models fitted by restricted maximum likelihood (REML), with effect sizes nested within studies. Separate models were conducted for hospitalization, bed rest, spaceflight, posthospitalization and postbed rest. For controlled conditions, standardized mean differences were analysed, with muscle mass models adjusted for measurement category (muscle volume, muscle area, or total lean mass; reference = total lean mass). Spaceflight effects were analysed using standardized mean change (SMC) from pre–postdata with small‐sample correction, and sensitivity analyses were performed across plausible pre–postcorrelations (*r* = 0.5–0.9). All analyses were conducted in Stata/SE 14.0.

The studies were analysed separately by hospitalization, bed rest and spaceflight mission. Muscular strength and power outcomes were divided into the following subgroups: upper‐body muscle strength (handgrip test), lower body muscle strength (1‐RM test) and lower body muscle power (isokinetic test). Muscle mass outcome was obtained from image‐based assessment (e.g., DXA and ultrasound) and was divided into the following subgroups: muscle volume, muscle area and total lean mass. In this sense, ES of muscular strength and muscle mass were calculated using preintervention and postintervention means, SD pooled and sample size. The criterion for statistical significance was set at *p* < 0.05 in a *Z*‐test analysis to examine whether ES was significantly different from zero using a random‐effect model. Study heterogeneity was evaluated using the *I* ^2^ statistic, and Cochrane's Q. Values of *I* ^*2*^ higher than 50% and 75% were considered moderate and high heterogeneity. For Cochrane's Q, significant heterogeneity exists when the Q value exceeds the estimate's degrees of freedom (df). Moreover, the asymmetry of the funnel's plot was tested using Eggers's test conducted in Stata/SE 14. (see Data S5 and Table S1). Sensitivity analyses were performed by excluding one trial at a time according to the RoB to test the robustness of the pooled results. Forest plots were generated to illustrate the between‐study‐level ES ± 95% CI.

## 3 Results

**FIGURE 1:**
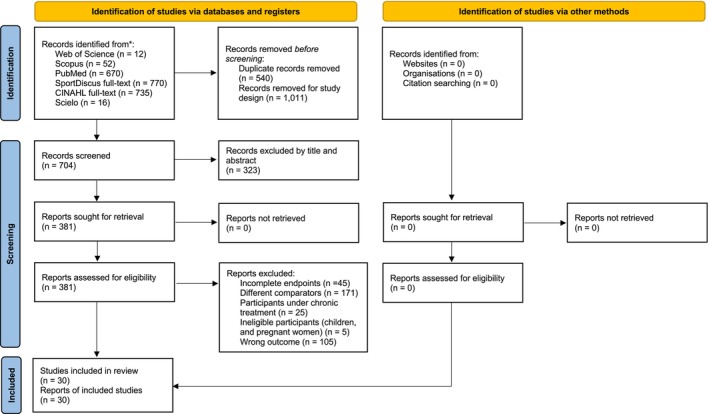
Flow diagram of the study selection process.

A total of 2255 (950 studies retrieved in May 2021 and 1305 new studies retrieved in March 2025) as documented in the PRISMA flow diagram (Figure 1), 704 records were then assessed by title and abstract, 381 full‐text records were assessed according to PICOS eligibility criteria, and a total of 30 studies were included in the meta‐analysis (Figure 1). Seventy‐five per cent of included studies were qualitatively classified as low RoB (see Data S4).

The participants' characteristics included in this meta‐analysis are presented in supplementary material (see Tables S2, S3, S4 and S5). A total of 1754 individuals (1161 males and 593 females, aged 24 to 88 years old), where 1013 (600 males and 413 females) were hospitalized due to acute medical illness (e.g., fall, heart failure and exacerbating chronic obstructive pulmonary disease) with ages ranging 58 to 87 years [36, 37, 38, 39, 40]; 207 (162 males and 45 females) were bedridden (e.g., forced bed rest to induce sarcopenia or microgravity simulation (bed rest head‐down)) with ages of 20 to 40 years [41, 42, 43, 44, 45, 46, 47, 48, 49, 50, 51, 52]; 206 (187 males and 19 females) astronauts were included with spaceflight experience aged between 40 to 55 years [11, 53, 54, 55, 56, 57, 58]; 214 individuals (90 males and 124 females) were recovering from hospitalization and engaged in exercise therapy as treatment aged between 60 and 87 years [59, 60, 61, 62]; and 24 males were recovering from bed rest and engaged in exercise therapy as treatment strategy, aged between 28 and 36 years [63]. The meta‐analysis was separated by hospitalization (5–15 days) [36, 37, 38, 39, 40], bed rest (30–90 days) [41, 42, 43, 44, 45, 46, 47, 48, 49, 50, 51, 52], spaceflight mission (160–240 days) [11, 53, 54, 55, 56, 57, 58, 64] and rehabilitation from hospitalization and bed rest (40–168 days) [59, 60, 61, 62, 63].

### 3.1 Exercise Intervention Characteristics

Hospitalization‐based exercise interventions involved multicomponent regimens that consisted of resistance training, gait retraining and balance training [36, 37, 38, 39, 40]. The resistance training (RT) component involved free weights, resistance machines and/or body weight for a range of movements (such as dorsiflexion, knee flexion and extension, hip flexion, elbow flexion and extension and shoulder flexion) with a resistance of 30%–60% of one‐repetition maximum (1‐RM) and 2–3 sets of 5–10 repetitions. Bed rest exercise intervention involved RT [41, 42, 44, 45, 46, 47, 48, 49, 50, 51, 56] and endurance exercise [51, 52]. Bed rest and microgravity simulation studies involving RT programmes included Smith machines, flywheel ergometers, or the Galileo space exercise device (20–40 min per session, 1–4 sets of 8–12 repetitions, 40%–80% of 1‐RM, 3 times per week). Studies investigating endurance training involved exercise (high‐intensity interval training and continuous exercise, 40%–80% of $\dot{V}$O_2peak_ and rating of perceived exertion (RPE) of 4–7 on the BORG scale). In‐flight space mission‐based exercise intervention involved systems specifically developed for exercise in space, the interim resistive exercise device (iRED) or advanced resistive exercise device (ARED), 150 min per session (90 min of resistance exercise (50%–85% of 1‐RM) and 60 min of cardiorespiratory exercise (75% of $\dot{V}$O_2peak_)), twice to three times per day during spaceflights [11, 53, 54, 55, 56, 57, 58, 64]. Exercise interventions as therapy to mitigate disused‐induced muscle atrophy involved mostly resistance exercise, such as flywheel resistance, which is a device that is gravity independent and utilizes inertia of rotating flywheels, weights equipment and elastic resistance band. These interventions involved two to three exercise sessions per week, 40%–70% of 1‐RM, 1–3 sets of 8–10 repetitions maximum.

### 3.2 Exercise Intervention Impact on Neuromuscular Strength

**FIGURE 2:**
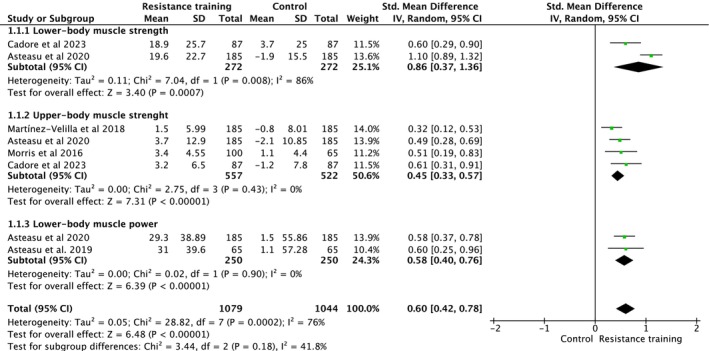
Meta‐analysis performed on the effects of exercise intervention as a preventive strategy on neuromuscular strength in individuals during hospitalization. Calculations are based on a random‐effects model. Results are expressed as effect size (ES) and 95% confidence intervals (95% CI).

Hospitalization‐based exercise intervention studies assessed lower body and upper‐body muscle strength and power (Figure 2). Overall, exercise performed during hospitalization increased muscle strength and power (ES = 0.60, 95% CI [0.42, 0.78], heterogeneity: *p* = 0.0002, *I* ^2^ = 76%, random‐effect model: *p* = 0.0007) (Figure 2). Subgroup analysis indicated a significant increase of lower body muscle strength (ES = 0.86, 95% CI [0.37, 1.36], heterogeneity: *p* = 0.008, *I* ^2^ = 86%, random‐effect model: *p* = *<* 0.0001), significant increase of upper‐body muscle strength (ES = 0.45, 95% CI [0.33, 0.57], heterogeneity: *p* = 0.430, *I* ^2^ = 0%, random‐effect model: *p* = *<* 0.0001) and significant increase of lower body muscle power (ES = 0.58, 95% CI [0.40, 0.76], heterogeneity: *p* = 0.900, *I* ^2^ = 0%, random‐effect model: *p* = *<* 0.0001) (Figure 2). Multilevel meta‐analysis accounting for within‐study dependence demonstrated a significant increase in muscle strength during hospitalization. The adjusted pooled effect remained significant after hierarchical modelling (ES = 0.58, 95% CI [0.19, 0.96] *p* = 0.003), confirming a robust association between hospitalization and muscle strength loss.

**FIGURE 3:**
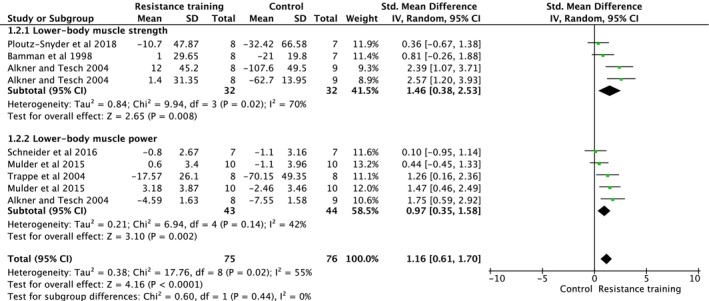
Meta‐analysis performed on the effects of exercise intervention as a preventive strategy on neuromuscular strength in individuals during bed rest. Calculation based on random‐effects model. Results are expressed as effect size (ES) and 95% confidence intervals (95% CI).

Bed rest and microgravity stimulation‐based exercise intervention studies assessed lower body muscle strength and power (Figure 3). Overall analysis indicated that exercise interventions increased muscle strength and power (ES = 1.16, 95% CI [0.61, 1.70], heterogeneity: *p* = 0.020, *I* ^2^ = 55%, random‐effect model: *p* = *<* 0.0001) (Figure 3). Subgroup analysis indicated a significant increase in lower body muscle strength (ES = 1.46, 95% CI [0.38, 2.53], heterogeneity: *p* = 0.020, *I* ^2^ = 70%, random‐effect model: *p* = 0.008) and lower body muscle power (ES = 0.97, 95% CI [0.35, 1.58], heterogeneity: *p* = 0.140, *I* ^2^ = 42%, random‐effect model: *p* = 0.002) (Figure 3). In multilevel REML models including correlated lower limb strength and power outcomes, no significant pooled effect was observed following bed rest (ES = 0.06, 95% CI [−1.31, 1.43], *p* = 0.93). Adjustment for within‐study dependence did not alter the conventional random‐effects analyses.

**FIGURE 4:**
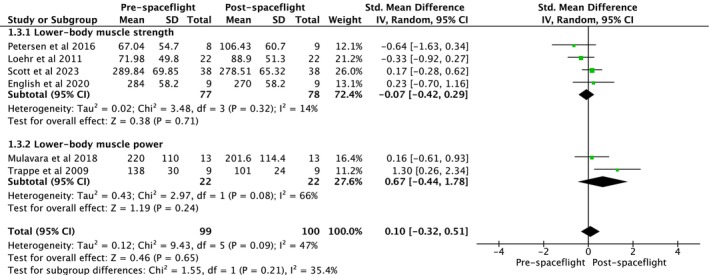
Meta‐analysis performed on the effects of exercise intervention as a preventive strategy on neuromuscular strength in individuals during spaceflight mission. Calculation based on random‐effects model. Results are expressed as effect size (ES) and 95% confidence intervals (95% CI).

In‐flight‐based exercise intervention studies assessed lower body muscle strength and power (Figure 4). Overall analysis indicated that exercise intervention had no impact on lower body muscle strength and power (ES = 0.10, 95% CI [−0.32, 0.51], heterogeneity: *p* = 0.090, *I* ^2^ = 47%, random‐effect model: *p* = 0.650) (Figure 4). Subgroup analysis indicated no effect of exercise intervention on lower body muscle strength (ES = −0.07, 95% CI [−0.42, 0.29], heterogeneity: *p* = 0.320, *I* ^2^ = 14% and random‐effect model: *p* = 0.710). Although no effect on lower body muscle power, exercise intervention resulted in a moderate effect size (ES = 0.67, 95% CI [−0.44, 1.78], heterogeneity: *p* = 0.080, *I* ^2^ = 66%, random‐effect model: *p* = 0.240) (Figure 4). Muscle mass outcomes were analysed using SMC from pre–postcomparisons. The pooled SMC indicated no significant overall change in muscle mass following spaceflight (ES g = 0.002, 95% CI [−0.077, 0.080], *p* = 0.966). Sensitivity analyses assuming different pre–postcorrelations (*r* = 0.5, 0.7 and 0.9) yielded consistent estimates, confirming robustness of the findings.

**FIGURE 5:**
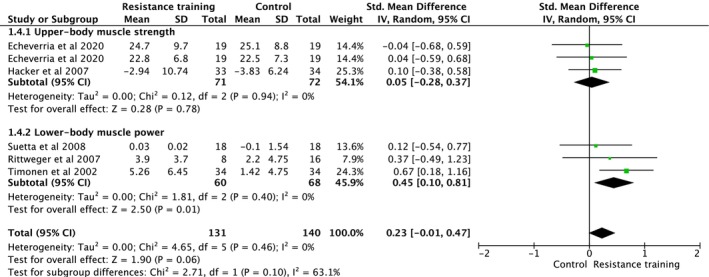
Meta‐analysis performed on the effects of exercise intervention as a therapeutical strategy on neuromuscular strength in individuals after hospitalization and bed rest. Calculation based on random‐effects model. Results are expressed as effect size (ES) and 95% confidence intervals (95% CI).

Studies investigating exercise interventions as therapy to recover from disuse‐related muscle atrophy assessed upper‐body muscle strength and lower body muscle power (Figure 5). Overall analysis indicated that exercise intervention showed a trend to increases of muscle strength and power (ES = 0.23, 95% CI [−0.01, 0.47], heterogeneity: *p* = 0.460, *I* ^2^ = 0%, random‐effect model: *p* = 0.060) (Figure 5). Subgroup analysis indicated no change on upper‐body muscle strength (ES = 0.05, 95% CI [−0.28, 0.37], heterogeneity: *p* = 0.940, *I* ^2^ = 0%, random‐effect model: *p* = 0.780). However, there was a significant increase of lower body muscle power (ES = 0.45, 95% CI [0.10, 0.81], heterogeneity: *p* = 0.400, *I* ^2^ = 0%, random‐effect model: *p* = 0.010) (Figure 5). Pooled effects estimated using multilevel models with random intercepts indicated no significant changes on upper‐body muscle strength and lower body muscle power (ES = 0.30, 95% CI [−0.34, 0.95], *p* = 0.361).

### 3.3 Exercise Intervention Impact on Muscle Mass

**FIGURE 6:**
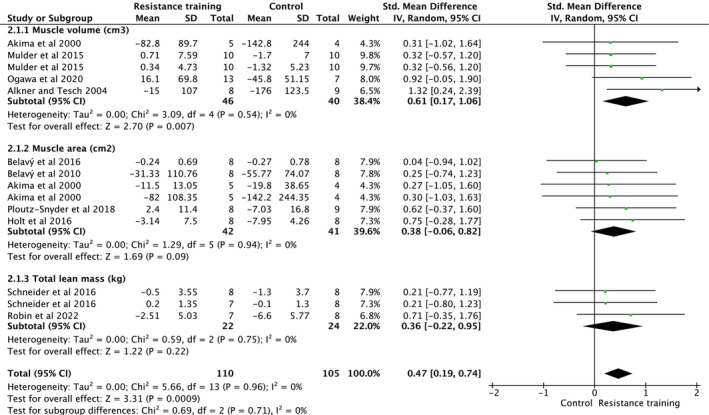
Meta‐analysis performed on the effects of exercise intervention as a preventive strategy on muscle mass in individuals during bed rest. Calculation based on random‐effects model. Results are expressed as effect size (ES) and 95% confidence intervals (95% CI).

Bed rest and microgravity stimulation‐based exercise intervention studies assessed muscle volume (cm^3^), muscle area (cm^2^) and total lean mass (kg) (Figure 6). Overall analysis indicated that exercise intervention significantly increased muscle mass (ES = 0.47, 95% CI [0.19, 0.74], heterogeneity: *p* = 0.960, *I* ^2^ = 0%, random‐effect model: *p* = 0.009) (Figure 6). Subgroup analysis showed a significant increase of muscle volume (cm^3^) (ES = 0.61, 95% CI [0.17, 1.06], heterogeneity: *p* = 0.540, *I* ^2^ = 0%, random‐effect model: *p* = 0.007). However, there was no effect of exercise intervention on muscle area (cm^2^) (ES = 0.38, 95% CI [−0.06, 0.82], heterogeneity: *p* = 0.940, *I* ^2^ = 0%, random‐effect model: *p* = 0.090) and on total lean mass (kg) (ES = 0.36, 95% CI [−0.22, 0.95], heterogeneity: *p* = 0.750, *I* ^2^ = 0% and random‐effect model: *p* = 0.220). Multilevel REML models adjusted by muscle mass measurement type (muscle volume, muscle area and total lean mass; reference = total lean mass) revealed measurement‐dependent differences in effect magnitude. Compared with total lean mass, muscle volume and muscle area exhibited significantly increase (ES = 0.39, 95% CI [0.06, 3.51], *p* = 0.042). The random intercept variance was significant (*p* = < 0.001), indicating meaningful between‐study heterogeneity.

**FIGURE 7:**
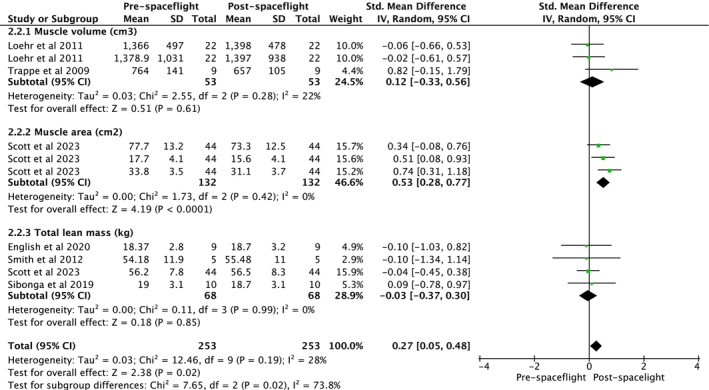
Meta‐analysis performed on the effects of exercise intervention as a preventive strategy on muscle mass in individuals during spaceflight mission. Calculation based on random‐effects model. Results are expressed as effect size (ES) and 95% confidence intervals (95% CI).

Studies assessing the impact of exercise interventions during spaceflight evaluated muscle volume (cm^3^), muscle area (cm^2^) and total lean mass (kg) (Figure 7). Overall analysis indicated a significant increase in muscle mass in astronauts (ES = 0.27, 95% CI [0.05, 0.48], heterogeneity: *p* = 0.190, *I* ^2^ = 28%, random‐effect model: *p* = 0.020) (Figure 7). Subgroup analysis indicated no changes in muscle volume (cm^3^) (ES = 0.12, 95% CI [−0.33, 0.56], heterogeneity: *p* = 0.280, *I* ^2^ = 22%, random‐effect model: *p* = 0.610) and total lean mass (kg) (ES = −0.03, 95% CI [−0.37, 0.30], heterogeneity: *p* = 0.990, *I* ^2^ = 0%, random‐effect model: *p* = 0.850). On the other hand, exercise intervention increased muscle area (cm^2^) (ES = 0.53, 95% CI [0.28, 0.77], heterogeneity: *p* = 0.420, *I* ^2^ = 0%, random‐effect model: *p* = < 0.0001) (Figure 7). Because no parallel control groups were available, muscle mass outcomes were analysed using SMC from pre–postcomparisons. The pooled SMC indicated no significant overall change in muscle mass following spaceflight (ES = 0.002, 95% CI [−0.077, 0.080], *p* = 0.966). Sensitivity analyses assuming different pre–postcorrelations (*r* = 0.5, 0.7, and 0.9) yielded consistent estimates, confirming robustness of the findings.

## 4 Discussion

We present the first meta‐analytical study to investigate the impact of exercise interventions as both preventive and therapeutic strategies to mitigate skeletal muscle disuse associated with hospitalization, bed rest and spaceflight. Our primary analyses indicate that exercise performed as a preventive strategy during hospitalization and bed rest significantly improves muscle strength and power, whereas no consistent benefits are observed during spaceflight missions. Importantly, multilevel meta‐analyses accounting for within‐study dependence confirmed a robust beneficial effect of exercise on muscle strength during hospitalization but attenuated the magnitude of pooled effects during bed rest, indicating that previously reported large effects were partly driven by correlated outcomes. In contrast, exercise interventions applied therapeutically after disuse showed a trend toward improved muscle strength and power in conventional analyses; however, these effects were no longer statistically significant in multilevel models, highlighting substantial heterogeneity and limited power. Notably, subgroup analyses revealed a significant improvement in lower body muscle power, suggesting that limb‐ and outcome‐specific adaptations may persist even when overall pooled effects are modest. Furthermore, preventive exercise interventions during bed rest and spaceflight significantly preserved or increased muscle mass, although standardized mean change analyses in spaceflight indicated no consistent overall change. These findings are clinically relevant, as neuromuscular strength, muscle power and muscle mass are key predictors of survival and functional outcomes in disuse‐induced muscle atrophy (Table 1). Collectively, our results underscore that exercise—particularly when implemented preventively—plays a critical role in attenuating the deleterious effects of disuse, while postdisuse interventions may offer more limited and outcome‐specific benefits. This nuanced interpretation emphasizes the importance of early implementation and appropriate outcome selection when prescribing exercise as a countermeasure for disuse‐induced muscle atrophy.

**TABLE 1:** Summary of overall effect of preventive and therapeutic exercise on outcomes changed in hospitalized older individuals, bed rest young adults and astronauts during spaceflight missions.

Outcomes	Hospitalization	Bed rest	Spaceflight mission
*Preventive exercise*			
Muscle strength and power	↑	↑	↔
Muscle mass	—	↑	↑
*Therapeutic exercise*			
Muscle strength and power	↔	↔	—
Muscle mass	—	↑	↑

*Note:* ↑ increase and ↔ unchanged.

An important consideration when interpreting these findings is the possibility of sex‐specific responses to disuse and exercise interventions [65]. Although both men and women were represented in hospitalization and bed rest studies, most trials were not designed or sufficiently powered to allow robust sex‐stratified analyses. Biological differences related to muscle morphology, hormonal environment, neuromuscular activation and anabolic sensitivity are likely to influence both the extent of muscle loss during disuse and the responsiveness to exercise‐based countermeasures [66, 67]. Available evidence indicates that women may experience proportionally greater declines in muscle strength during unloading, whereas men often show larger absolute reductions in muscle mass; however, adaptive responses to exercise may also differ between sexes, particularly in older populations [68, 69]. These challenges are even more pronounced in spaceflight research, where small‐sample sizes and the limited inclusion of female astronauts constrain meaningful sex‐specific interpretation [70]. Taken together, while our results support exercise as a preventive and therapeutic strategy across disuse contexts, future studies should be explicitly designed to examine sex‐specific adaptations in order to refine intervention prescription and improve the generalizability of countermeasures for both men and women.

Critically ill individuals represent a major clinical challenge following prolonged intensive care treatment, as they frequently develop intensive care unit–acquired weakness, a condition associated with sepsis, multiorgan failure, glucocorticoid exposure, neuromuscular blocking agents, hyperglycaemia and prolonged immobility [71]. In this context, Nakanishi and colleagues [72] reported observational associations between participation in a short‐term (7 day) progressive resistance‐based physical therapy programme during ICU stay and higher measures of muscular strength (≈12% handgrip) and upper‐ and lower body muscle mass (≈12% and ≈15%, respectively) in older men and women (~70 years). However, as this study was observational in nature, it does not allow inference of causality, nor was it designed to evaluate effects on clinical outcomes such as survival.

More recently, studies have introduced head‐down tilt bed rest as an experimental model to replicate microgravity and simulate spaceflight [73, 74, 75, 76]. Research has provided unique insights into the physiology of nonweight‐bearing and deconditioning associated with spaceflight through microgravity simulation, extensively reported by Pavy‐Le and colleagues [73, 74, 75, 76, 77] and later by Hargens and colleagues [73]. Several studies have investigated the effect of exercise therapy as a potential intervention for astronauts to maintain their health, muscle structure and function during and after spaceflight. However, pragmatic implementation of such interventions has presented challenges due to difficulty assessing astronauts during spaceflight, and the cost of equipment adapted for the space vehicles and environment [18]. Over many decades, the National Aeronautics and Space Administration (NASA) has dedicated financial resources and research to investigate how to attenuate the harmful effects of long‐duration spaceflight, including exercise therapy [53]. Currently, astronauts may engage in two protocols of training during space missions, as follows: iRED and ARED that involve upper‐ and lower body exercise [53]. Although most studies are not overly favourable, given that neither iRED nor ARED exercise interventions (3–6 sessions per week) during long‐duration spaceflight (~180 days) seem to attenuate the loss of neuromuscular strength and functional capacity, or preserve muscle and bone mass [56, 57]; our subgroup analysis showed a significant increase in muscle area of astronauts after returning from spaceflight.

Benefits of exercise therapy on muscle mass and function may be attributed to several mechanisms, such as muscle size and architecture, metabolism and protein regulation [78] combined with neural adaptations such as motor unit recruitment, rate coding, rate of onset and intra‐ and intermuscular coordination [4, 7]. Mechanical loading is considered a pivotal stimulus to mitigate the harmful effect of muscle disuse and subsequent bone loss that links human exercise to skeletal muscle hypertrophy and immunometabolic response [4, 7, 24, 78]. High forces distinguish hypertrophy‐inducing resistance exercise from low‐load endurance exercise that triggers little or no hypertrophy [78, 79]. Previous studies report that mechanical loading does not necessarily need to be heavy to promote muscle hypertrophy, with loads as low as ~30% 1‐RM until concentric neuromuscular failure sufficient to trigger hypertrophic response [78]. Lasevicius and colleagues [80] investigated 12 weeks of resistance exercise using leg extension and elbow extension with one leg or arm at 20% 1‐RM and then either 40%, 60% or 80% with the opposite leg or arm. The authors reported that at least 40% 1‐RM with sets performed to failure caused similar hypertrophy to the higher load conditions. Furthermore, in untrained individuals, even submaximal aerobic training, low mechanical load exercise [80, 81] or very low loads such as 16% 1‐RM increase muscle protein synthesis [82]. In summary, mechanical load represents a critical driver stimulating muscle hypertrophy. However, this intensity of RT may not be sufficient to drive recovery after disuse‐induced skeletal muscle atrophy when loads are miscalculated and low volume is applied.

Life on Earth evolved in an environment where gravity is constantly mechanically loading all organisms, and living beings and their cells have not only developed mechanical structures such as the muscles, skeleton and cytoskeleton to withstand or overcome the pull of gravity but also a plethora of sensors that detect the mechanical stimulus [4, 7]. Cumulative exercise therapy sessions present a pleiotropic effect, leading to several morphological and phenotypical skeletal muscle adaptations induced by these sensors [83]. However, even with a large volume of resistance exercise, astronauts during long‐duration spaceflight still experience a decline in neuromuscular strength (~28%) and muscle mass (~15%) [10, 21]. Therefore, both physiological and mechanical effects exposed by microgravity might interfere with expected gains in neuromuscular function and muscle mass; the mechanisms of which remain unknown. While we are confident that exercise as a preventive strategy is promising to improve neuromuscular strength during hospitalization, bed rest or spaceflight, some limitations in this meta‐analysis must be presented. Although exercise therapy has emerged as a strategy to improve skeletal muscle quality, size and function in a normal gravity environment, investigating the effect of interventions during spaceflight is limited by fewer studies of small‐sample sizes and nonrandomized designs. In addition, caution is warranted when interpreting the overall findings, given the wide variation in exercise intensities, participant ages and the differing durations of both disuse and exercise interventions across studies. Nonetheless, a strength of this meta‐analysis is that most studies demonstrated a moderate to low risk of bias. Another strong point is its pioneering focus on evaluating exercise as a preventive strategy during hospitalization, bed rest or spaceflight—contexts in which most participants completed the interventions with high compliance and evident benefit.

## 5 Conclusion

This meta‐analysis demonstrates that exercise, when employed as a preventive strategy, effectively attenuates muscle strength loss and preserves muscle mass during hospitalization and bed rest, whereas no consistent benefits are observed during spaceflight. These effects remain robust after accounting for within‐study dependence using multilevel modelling. Exercise initiated after disuse shows potential improvements in muscle strength and power, which appear modest and fail to reach statistical significance in multilevel analyses, suggesting that recovery of muscle function may be slower, more variable or less responsive once disuse‐related impairments are established. Differences between conventional random‐effects and multilevel estimates further emphasize the importance of appropriately accounting for outcome dependence and heterogeneity when synthesizing disuse‐related exercise data. Future high‐quality randomized controlled trials with standardized outcome reporting, adequate sample sizes and clearly defined exercise prescriptions are required to optimize the timing, modality and dose of exercise for clinical rehabilitation and aerospace applications.

## Conflicts of Interest

The authors declare no conflicts of interest.

## Supporting information


**Table S1:** Distribution of studies across conditions and outcomes for Egger's regression analyses.

**Table S2:** Studies characteristics of hospitalized individuals.

**Table S3:** Study characteristics of individuals during bed rest.

**Table S4:** Studies characteristics of astronauts during spaceflight mission.

**Table S5:** Studies characteristics of individuals recovering from disuse condition.

**Data S1:** Supporting information.

**Data S2:** Supporting information.

**Data S3:** Supporting information.

**Data S4:** Supporting information.

**Data S5:** Supporting information.

**Data S6:** Supporting information.

## Data Availability

The data and materials analysed during the present study are available from the corresponding author on reasonable request.
