# Supplementary material for: Disuse‐Induced Muscle Atrophy and Muscle Weakness From Hospitalization to Spaceflight: Exercise Succeeds in Prevention and Treatment—A Meta‐Analysis
Source: J Cachexia Sarcopenia Muscle. 2026 Apr 15;17(2):e70259. doi: 10.1002/jcsm.70259 (PMC13080877; doi:10.1002/jcsm.70259)
Supplement: Supplementary file 1 — Table S1: Distribution of studies across conditions and outcomes for Egger's regression analyses. [file JCSM-17-e70259-s010.pdf]

Table S1. Distribution of studies across conditions and outcomes for Egger's regression analyzes.

Condition	Outcome	Number of studies (k)	Coefficient	Standard error	95% CI	Egger test
Hospitalization	Muscle strength/power	8	0.863	2.328	-4.834007 to 6.561282	p = 0.723*
Bed rest	Muscle strength/power	9	9.700	2.045	4.863739 to 14.53661	p = 0.002*
Post-hospitalization/bed rest	Muscle strength/power	6	4.041	2.907	-4.029877 to 12.11345	p = 0.237*
Bed rest	Muscle mass	11	4.690	3.458	-3.13245 to 12.51282	p = 0.208

Note: * represents small-study effects (< 10 k). Given the absence of control groups and the limited number of studies with complete variance information, Egger's regression test was not interpreted for these syntheses.
