# Supplementary material for: Disuse‐Induced Muscle Atrophy and Muscle Weakness From Hospitalization to Spaceflight: Exercise Succeeds in Prevention and Treatment—A Meta‐Analysis
Source: J Cachexia Sarcopenia Muscle. 2026 Apr 15;17(2):e70259. doi: 10.1002/jcsm.70259 (PMC13080877; doi:10.1002/jcsm.70259)
Supplement: Supplementary file 2 — Table S2: Studies characteristics of hospitalized individuals. [file JCSM-17-e70259-s011.pdf]

Table S1. Studies characteristics of hospitalized individuals.

Author/Year	Age/Sex	Sample	Objective	Duration (days)	Exercise protocol	Measurement	Main outcome
Asteasu et al. 2019	87 yrs (Male/Female)	N = 370 (UC=185 and RE=185)	To examine the individual response of acutely hospitalized patients to usual care and to physical exercise on functional capacity, muscle strength, and cognitive function.	5-7	Twice a day (morning/evening), 20 min per session, 2-3 sets of 8-10 reps, 30-60% 1-RM. Following exercises (squats rising from a chair, leg press, and bilateral knee extension) and one involving the upper body musculature (seated bench 'chest' press).	Handgrip (muscular strength), SPPB and gait speed (function capacity).	Handgrip = ↑ 47.05 % in RE group compared with UC group.<br>SPPB = ↑ 70.2% in RE group compared with UC group.<br>Gait speed = ↑ 3.10% on gait speed in RE group compared with UC group
Asteasu et al. 2020	87 yrs (Male/Female)	N = 370 (UC=185 and RE=185)	To examine the individual response of acutely hospitalized patients to usual care and to physical exercise on functional capacity, muscle strength, and cognitive function.	5-7	Twice a day (morning/evening), 20 min per session, 2-3 sets of 8-10 reps, 30-60% 1-RM. Following exercises (squats rising from a chair, leg press, and bilateral knee extension) and one involving the upper body musculature (seated bench 'chest' press).	Maximal dynamic strength (i.e. leg-press, chest-press, and knee extension exercises) and maximal isometric knee extensors and hip flexors strength. Muscle power output at submaximal and maximal loads.	1-RM leg press = ↑ 19.6% in RE group compared with UC group.<br>1-RM knee extension = ↑ 9.4 % in RE group compared with UC group.<br>1-RM chest press ↑ 5.7% on 1-RM in RE group compared with UC group.<br>Maximal isometric strength = ↑ 22.1% knee extension and ↑ 20.8% hip flexion in RE group compared with UC group.<br>Peak of power 60% 1-RM = ↑ 30.1 w, 75% 1-RM = ↑ 35.6 w, 100% 1-RM = ↑ 29 w
Martínez-Velilla et al. 2018	87 yrs (Male/Female)	N = 370 (UC=185 and RE=185)	To assess the effects of an innovative multicomponent exercise intervention on the functional status of this patient population.	5-7	Twice a day (morning/evening), 20 min per session, 2-3 sets of 8-10 reps, 30-60% 1-RM. Following exercises (squats rising from a chair, leg press, and bilateral knee extension) and one involving the upper body musculature (seated bench 'chest' press).	SPPB, Barthel Index and handgrip.	SPPB= ↑ 2.2 points.<br>Barthel Index = ↑ 6.9 points; Handgrip = ↑ 1.5 kg in RE group compared with UC group.
Morris et al. 2016	58 yrs (Male/Female)	N = 300 (UC=150 and RE=150)	To compare standardized rehabilitation therapy to usual ICU care in acute respiratory failure.	10	Tree sessions per day, 3 sets of 8 reps, following exercise included dorsiflexion, knee flexion and extension, hip flexion, elbow flexion and extension, and shoulder flexion	SPPB, handgrip and dynamometer strength.	SPPB = ↔ in both groups, handgrip = ↔ in both groups, and dynamometer strength = ↔ in both groups.

Note. UC = Usual Care; RE = Rehabilitation Therapy; SPPB = Short Physical Performance Battery; ICU = Intensive Care Unit.
