# Supplementary material for: Disuse‐Induced Muscle Atrophy and Muscle Weakness From Hospitalization to Spaceflight: Exercise Succeeds in Prevention and Treatment—A Meta‐Analysis
Source: J Cachexia Sarcopenia Muscle. 2026 Apr 15;17(2):e70259. doi: 10.1002/jcsm.70259 (PMC13080877; doi:10.1002/jcsm.70259)
Supplement: Supplementary file 3 — Table S3: Study characteristics of individuals during bed rest. [file JCSM-17-e70259-s004.pdf]

Table S2. Study characteristics of individuals during bed rest.

Author/Year	Age/Sex	Sample	Objective	Duration (days)	Exercise protocol	Measurement	Main outcome
Akima et al. 2000	18-30 yrs (Male)	N = 9 (RE = 5 and Control = 4)	To investigate the effects of resistance training on the morphological and functional properties of human lower limb muscles during 20 days of 6° head-down-tilt BR.	20	Isometric leg-press exercises were performed: 3 s x 30 repetitions (30 s rest between repetitions) daily for 20 days during the bed-rest period.	Serial axial magnetic resonance images were taken from the right thigh and leg muscles, and muscle volume, muscle length, and fiber length were estimated. The PCSAs of the knee extensor, knee flexor, ankle plantar flexor, and ankle dorsiflexor (tibialis anterior) muscle groups were determined as muscle volume multiplied by the cosine of the angle of fiber pennation divided by fiber length. Maximum voluntary contraction during knee extension was measured.	PCSAs knee extensors = ↔ in RE group compared to Control group. PCSAs knee flexor and plantar flexor volume = ↓ in RE and Control group. Maximum voluntary contraction = ↑ RE group compared to Control group.
Alkner and Tesch 2004	26-41 yrs (Male)	N = 17 (RE = 9 and Control = 8)	To investigate the effects of plantar flexor and knee extensor RE on muscle size and function in 17 healthy men subjected to 90 days 6 head-down-tilt BR.	90	The RE program consisted of coupled maximal concentric and eccentric actions in the supine squat (4 sets of 7 repetitions) and calf press (4x4) every third day employing a gravity-independent flywheel ergometer.	Prior to, and following BR, muscle volume was assessed using magnetic resonance imaging. Similarly, muscle strength and power and surface electromyographic activity were determined during maximal actions using flywheel ergometer or isokinetic dynamometry.	Knee extensors volume = ↑ in Control group compared to RE group, plantar flexors volume = ↔ in RE group compared to Control group and lower-body strength and power = ↔ in RE compared to Control group.

Bamman et al. 1998	23-40 yrs (Male)	N = 17 (RE = 8 and Control = 8)	To test the efficacy of a concentric-eccentric constant resistance exercise countermeasure against the effects of BRU on knee extensor maximum voluntary strength, myofiber size, MHC distribution, and neural drive.	14	Concentric-eccentric constant-resistance leg-press exercise, once a day, 5-sets, 8 repetitions, 80-85% 1-RM for 14 days.	Muscular strength (1-RM), Maximal voluntary isometric strength and neural activation, vastus lateralis muscle cross-sectional area and MHC.	CSA = ↑ 2.92% in RE group compared with Control group; 1-RM leg press = ↑ 0.48% in RE group compared with Control group, knee extension = ↓ 11.25% in RE group compared with Control group, knee flexion = ↓ 17.54% in RE group compared with Control group, MHC (Type I = ↑ 8.62% in RE group compared with Control group, Type IIa = ↓ 4.60% in RE group compared with Control group, Type IIx = ↓ 5.76% in RE group compared with Control group).
Belavý et al. 2010	20-45 yrs (Male)	N = 17 (RE = 8 and Control = 8)	To evaluate the effect of short-duration, high-load resistive exercise, with and without whole body vibration on lumbar muscle size, intervertebral disk and spinal morphology changes, and low back pain incidence during prolonged BR.	60	Hight-load exercise includes concentric-eccentric phase 4:4 s, 70-80% 1-RM, one-set, repetitions until failure, five minutes of rest between exercises, as following bilateral squats, single leg heel raises, double leg heel raises, back and toe raises at Galileo Space exercise device, three times per week during 60 days of BR.	Cross sectional area of multifidus, erector spinae, quadratus lumborum and psoas muscle (magnetic resonance image).	CSA = ↑ 56% on multifidus muscle, ↑ 38% on erector spinae muscle, ↑ 91.3% on quadratus lumborum and ↑ 189.4% on psoas muscle in RE group compared with Control group.
Belavý et al. 2016	26-41 yrs (Male)	N = 16 (RE = 8 and Control = 8)	To investigate atrophy in the deep abdominal muscles, spinal extensors, and the effect of high-load resistive exercise with and without whole-body vibration after 60 days of strict BR.	60	Training was performed 3 days per week during the BR phase. After a short warm-up, the after exercises were performed on the Galileo Space exercise device (Novotec Medical GmbH, Pforzheim, Germany; Fig. 1): bilateral squats (~75%–80% of pre–BR maximum voluntary contraction.	Extensor musculature, the erector spinae and multifidus were imaged additionally on days 1, 2, 3, 4, 5, 6, 7, 8, 9, 10, 11, 13, and 14 of BR. Lumbar spine length was measured at every testing session.	Transversus abdominis thickness = ↔ in RE group compared with Control group. Extensor and spinal lengthening = ↔ in RE group compared with Control group.
Mulder et al. 2015	24-34 yrs (Male)	N = 30 (RE = 10, Stand=10 and Control = 10)	To evaluate the effectiveness of a short and versatile daily exercise regime, named locomotion replacement training, to maintain muscle size, isometric strength, power, and endurance capacity of the leg muscles following 5 days of head-down tilt BR.	5	Subjects performed three blocks: block one consisted of 20 bilateral heel raises, 20 squats (90°) and 4 sets of 6 reactive jumps; block two consisted of 2 × 12 unilateral heel raises, 12 deep squats (60°) and jumping as above. A Smith Machine with fixed rails (PTS-1000 Dual Action Smith™ Cage, Hoist Fitness Systems, San Diego, USA) was used to guide the heel raise and squat exercises. Squats and heel raises were performed against body weight plus the additional weight of the barbell (15 kg). The reactive jumps and the cross hopping (left–right-left–right, etc.) exercises were performed without Smith	Muscle size, muscle function and blood analysis.	Knee extensors muscle size = ↑ 2.49%, maximum voluntary contraction = ↑ 13.50% in RE group compared with Control group. Plantar flexors muscle size = ↑ 1.48%, maximum voluntary contraction = ↑ 9.85% in RE group compared with Control group. Absolute nitrogen balance (cumulative intake − urinary excretion) = ↑ 35.70% in RE group compared with Control group.

					Machine. The reactive jumps were performed with the ball of the foot (heels not touching the ground) at ~3 repetitions per second separated by 15-s rest every six jumps. Cross hopping was performed continuously for 3 min at a frequency of 1.3 repetitions per second.		
Ogawa et al. 2020	24-38 yrs (Male)	N = 16 (RE = 8 and Control = 8)	To investigate the effects of 8 weeks of BR, with or without resistance exercise intervention, on the volumes of muscle tissue and the intramuscular, intermuscular, and subcutaneous adipose tissues of the thigh.	60	Overall, the lower quadrant regions of the body were targeted with exercise (3 days per week) during BR. Bilateral squats (from 10° to 90° knee flexion and back with the concentric and eccentric phases for 4 s each) were performed during the first training session at 75% and after the second training session at 80% at the most; the participants performed the exercise continuously until exhaustion. From the third training session, the force level increased by 5% in each session until the participant could only perform eight repetitions. In the subsequent sessions, if the participant improved in such a way that they could perform more than 10 repetitions in two adjoining sessions, the force level was increased by 5% again.	Consecutive axial magnetic resonance images were obtained before and after the BR. Using these images, the volumes of the muscle tissue and the intramuscular adipose tissue, intermuscular adipose tissue, and subcutaneous adipose tissue of the thigh were evaluated.	Volumes of the muscle tissue and the intramuscular adipose tissue, intermuscular adipose tissue, and subcutaneous adipose tissue = ↔ in RE group compared with Control group.<br>Thigh muscle tissue volume = ↔ in RE group compared with Control group.
Ploutz-Snyder et al. 2018	30-43 yrs (Male/ Female)	N = 18 (RE = 8 and Control = 9)	To investigate the safety and effectiveness of a new integrated aerobic and resistance exercise training prescription (SPRINT) using two different sets of exercise equipment: a suite of large International Space Station–like exercise equipment similar to what is found on the International Space Station and a single device with aerobic and resistance exercise capability in the spaceflight analog of BR.	70	Subjects performed aerobic exercise 6 days per week and resistance exercise 3 days per week using the ‘‘SPRINT’’ protocol. Aerobic exercise sessions consisted of alternating days of continuous cycle exercise for 30 min at 75% of VO_2peak (3 days per week) with interval treadmill sessions of 30 sec, 2-min, or 4-min intervals (3 days per week) at nearly maximal intensity.<br>Resistance training was trained 3 days per week and consisted of three sets of each of four supine lifts (squat, leg press, unilateral leg curl, and heel raise); squats and leg press were each performed using a standard shoulder-width stance, single-leg stance, or wide-leg stance on a rotating basis.	VO_2peak, ventilatory threshold, cardiac morphology and function, muscle mass and strength/power, and bone health were assessed before and after BR.	VO_2peak = ↔ in RE group compared with Control group. Leg press total work = ↔ in RE group compared with Control group. Isokinetic upper and lower leg strength = ↔ in RE group compared with Control group. Vertical jump power = ↔ in RE group compared with Control group. Maximal jump height = ↔ in RE group compared with Control group.

Robin et al. 2022	30-34 yrs (Male)	N = 22 (RES = 7, RUN = 7 and Control = 8)	To compare the effectiveness of two exercise countermeasure programs, running and resistance training, applied separately, for counteracting cardiovascular deconditioning induced by 90-day head-down BR.	90	The subjects ran continuously at about 70%–80% of their VO_2peak (80%–89% of their heart rate max) for 30min three times a week (Monday, Wednesday, and Saturday). In addition, once a week (Friday), they performed a high intensity run over 4 sets of 4 min (90% VO_2peak) followed by 3min of active rest (40% VO_2peak). The subjects exercised for 45 min three times a week (Monday, Wednesday, and Friday). They performed squats, heel raises, shoulder shrugs, and curls at 60%–80% of maximal voluntary contraction in 3 sets of 10–12 repetitions. For squats, the knee flexion angle was from~90° to ~170°, and repetition of concentric to eccentric contraction was about 2–3 s.	VO_2peak, orthostatic tolerance, continuous electrocardiogram and blood pressure, body composition, and leg circumferences were measured.	VO_2peak = (−26% in CON, −15% in RES, and −4% in RUN).<br>Lean mass = (−10% in CON vs. −5% to 6% in RES and RUN).<br>Thigh circumference (−7% in Control, −2% in RES, and −0.6% in RUN).<br>Calf circumference = (−10% in Control vs. −7% in RES vs. −5% in RUN).
Schneider et al. 2016	25 yrs (Male/Female)	N = 30 (RE=15 and Control = 15)	To evaluate the effectiveness of an LBNPEX protocol for preventing losses in leg lean tissue mass, strength, and endurance during 30 days of 6° head-down tilt BR, a space flight analog. Male and female monogynous twins were studied before and after BR, with one serving as a control and the other performing the LBNPEX countermeasure.	30	Subjects performed 40 min of exercise 6 days/week. Target exercise intensities for this protocol consisted of 7 min at 40% of pre-BR VO_2max, 3 min at 60%, 2 min at 40%, 3 min at 70%, 2 min at 50%, 3 min at 80%, 2 min at 60%, 3 min at 80%, 2 min at 50%, 3 min at 70%, 2 min at 40%, 3 min at 60%, and 5 min at 40%. After completion of the exercise protocol, the lower body negative pressure chamber was maintained for 5 min while the subjects resisted the suction pressure against the vertical treadmill belt.	Muscle strength and endurance, and lean body mass	Muscle strength concentric ankle flexion = ↓ 7% (Male) and 5% (Female), ankle extension = ↓ 5% (Male) and ↑ 6% (Female), isometric ankle flexion = ↓ 12% (Male) and 10% (Female), isometric ankle extension = ↓ 4% (Male) and 11% (Female). Leg lean tissue mass = ↓ 3% (Male) and ↑ 2% (Female).
Trappe et al. 2004	29-34 yrs (Male)	N = 12 (RE = 6 and Control = 6)	To examine the high-intensity resistance training effect on myocellular function could be maintained during a long-term period of unloading.	84	The RE group trained on the flywheel ergometer every third day (2–3 days per week) beginning on day 5 of BR.<br>Each exercise session consisted of four sets of seven (4×7) maximal repetitions employing the supine (6 deg head-down tilt) squat exercise. Two minutes of rest was allowed between sets. Force, flywheel rotational velocity (work and power were calculated) and the knee joint angles were recorded during each training session.	Muscle strength, cross section area by Magnetic Resonance Imaging, biopsy and MHC determination.	Whole muscle quadriceps size = ↔in RE group, concentric peak force = ↑ 4% and peak power = ↑ 9% in RE group compared with control group. MHC I and IIa = ↔ in RE group.

Note. RE = Rehabilitation Therapy; BR = Bed Rest; PCSAs = Physiological Cross-Sectional Areas; BRU = Bed-Rest Unloading; MHC = Myosin Heavy Chain; CSA = Cross Sectional Area; LBNPEX = Lower Body Negative Pressure; VO_2peak = Peak oxygen uptake.
