# Supplementary material for: Disuse‐Induced Muscle Atrophy and Muscle Weakness From Hospitalization to Spaceflight: Exercise Succeeds in Prevention and Treatment—A Meta‐Analysis
Source: J Cachexia Sarcopenia Muscle. 2026 Apr 15;17(2):e70259. doi: 10.1002/jcsm.70259 (PMC13080877; doi:10.1002/jcsm.70259)
Supplement: Supplementary file 4 — Table S4: Studies characteristics of astronauts during spaceflight mission. [file JCSM-17-e70259-s001.pdf]

Table S3. Studies characteristics of astronauts during spaceflight mission.

Author/Year	Age/Sex	Sample	Objective	Duration (days)	Exercise protocol	Measurement	Main outcome
English et al. 2020	41-55 yrs (Male/Female)	N = 26 (RE = 9 and Control = 17)	To compare physiologic outcomes after ~6 months of long-duration spaceflight in crewmembers who performed exercise countermeasures consisting of either (1) lower intensity/higher volume exercise (6 d wk−1 resistance exercise and 6 d wk−1 aerobic exercise) or (2) high intensity/lower volume exercise (3 d wk−1 resistance exercise and 6 dwk−1 aerobic exercise).	180	Subjects in RE group completed high intensity interval aerobic exercise (3 d wk−1) and continuous aerobic exercise (3 d wk−1) on alternating days. Specifically, each of the three interval workouts was completed once per week (8 × 30-s intervals; 6 × 2-min intervals; and 4 × 4-min intervals); continuous aerobic exercise consisted of 30-min bouts at 75% VO_2peak. Aerobic intensities were initially established based on heart rate at a percentage of VO_2peak and heart rate response to exercise during preflight. Resistance training for RE followed an undulating periodized model and was performed on the same day as the continuous aerobic exercise daily (high volume=4 sets of 12 repetitions, moderate volume= 4 sets of 8 repetitions, low volume=4 sets of 6 repetitions).	Bone densitometry and muscle mass, muscle strength, muscle function and cardiorespiratory fitness.	Leg lean mass = ↓ 1.93%, leg press 1-RM = ↓ 9.69%, bench press 1-RM = ↓ 9.89%, sit and reach = ↑ 1.04%, cone test = ↓ 2.96% in RE group compared with Control group. VO_2peak = ↓ 3.77% and peak of heart rate = ↑ 1.14% in RE group compared with Control group.
Mulavara et al. 2018	42-52 yrs (Male/Female)	N=42 (ISS=13 and BR=19)	To determine the effects of spaceflight on functional task performance and to identify the key physiological factors contributing to their deficits.	180	ISS exercise protocol inflight crew time was made available to each crewmember 6 d/wk for 90 min of resistance exercise and 60 min of cardiovascular exercise daily. Resistance exercises were performed on the ARED and typically involved the upper body (e.g., upright row, bent over row/bicep curl) and lower-body (e.g., squat, deadlift, and heel raise) exercises. Cardiovascular exercise involved continuous or interval exercise using a cycle ergometer or a treadmill. For treadmill exercise, astronauts donned a harness and loaded in the range from 58% to 85% body weight using bungees according to an individual prescription to keep them in contact with the motorized treadmill and provide a ground reaction force at their feet.	Functional tests, muscular strength, plasma volumes test and neuromuscular drive.	Seat egress and walk test = ↑, recovery from fall/stand = ↑, object translation = ↑, jump down = ↑, ladder climb = ↔, hatch opening = ↔, activity board = ↔ from pre- to post spaceflight mission. Maximum isometric force lower body = ↔, lower body maximum power = ↓, lower body total work = ↓, maximum upper body isometric force = ↔, upper body maximum power = ↔, upper body total work = ↔ and central muscle activation capacity = ↔ from pre- to post spaceflight mission.
Loehr et al. 2011	28-40 yrs (Male/Female)	N = 20 (Free weight = 9 and ARED = 11)	To compare the musculoskeletal effects of resistance	180	Subjects performed squat, heel raise, and dead lift exercises 3 days per week for 16 weeks. Squat, heel raise, and	Bone mineral density, and vertical jump were assessed	Muscle strength = ↑ in both groups. Muscle volume = ↑ in both groups. Vertical jump

			exercise training using ARED with the effects of training with free weight.		dead lift strength (1-RM using free weight and ARED),	before, during, and after training. Muscle mass and bone morphology were measured before and after training.	height = ↑ in both groups. Lumbar spine bone mineral density = ↑ in both groups.
Petersen et al. 2016	40 yrs (Male)	N = 8 (ISS pre=2 and ISS post=6)	To provide a description of ESA's individualized approach to in-flight exercise countermeasures and an up-to-date picture of how exercise is used to counteract physiological changes resulting from μG-induced adaptation. Changes in the absolute workload for resistive exercise, treadmill running and cycle ergometry throughout ESA's eight LDMs are also presented, and aspects of pre-flight physical preparation and post flight reconditioning outlined.	190	First exercise sessions planned for the cycle ergometer. The first scheduled exercise bout (of a maximum of 1 h) is conducted no earlier than the second day after arrival on ISS, and this is followed by an increase in exercise time and loading up to the scheduled 2.5 h with 4–5 sessions per device each week in a periodic order. The intensity of the initial sessions is relatively low (e.g. 50–60 % of pre-flight capacity established in pre-flight training sessions) and increased subsequently per crew discretion. Training loads for resistance exercise are increased at a rate of 3–5 % per week and training loads are targeted toward 80 % or higher of individual maximal capacity established. Increasing focus on resistive exercise and treadmill running and the elimination of cycle ergometry. If possible, further increases in load are implemented, whilst ensuring good posture control during resistance training to avoid injury.	Muscle strength.	Muscle strength squat = ↑, heel raise = ↑, deadlift = ↑, bench press = ↑ from pre- to post spaceflight mission in RE group. Maximum vertical load = ↑, maximum running speed = ↑ and maximum cycle ergometry load = ↑ from pre- to post spaceflight mission in RE group.
Scott et al. 2023	41-53 yrs (Male/Female)	N = 46 (Pre-flight = 46 and post-flight = 46	To evaluate the effects of ISS exercise countermeasures on multisystem function.	226	Resistance exercise was prescribed 3-6 days per week, and aerobic exercise was prescribed 5-6 days per week consisted by performed the bench press 1-RM and leg press 1-RM. Treadmill exercise was modified from a commercial Woodway Path treadmill (Woodway, Waukesha, WI) to support walking and running exercise between 2.4 and 19.3 km·h^{-1}. The user is loaded via a shoulder and waist harness which is attached to bungee cords and terminally, the treadmill deck surface. Cycle ergometer operates similarly to a standard cycle ergometer providing workloads between 25 and 350W at pedal speeds from 30-120 revolutions per minute.	Muscle strength and size, and cardiorespiratory fitness.	Muscle strength = ↓ pre- to post-flight. Muscle size = ↓ pre- to post-flight. Cardiorespiratory fitness = ↓ pre- to post-flight.

Sibonga et al. 2019	45-47 yrs (Male)	N = 28 (Pre-ARED=18 and ARED=10)	Investigating if the beneficial effects on postflight aBMD were due to individual or combined effects of alendronate and ARED.	180	The exercise regimen was comprised of ARED resistance training and aerobic exercise (using a cycle ergometer or treadmill). In particular, the ARED device allows 17 different exercise configurations, including 4 types of squats, 3 types of deadlifts, 2 types of heel raise, 3 types of rows, as well as shrugs, shoulder press, bench press, bicep curl, and triceps extension.	Muscle mass.	Whole body lean mass = ↑ 0.35% and leg lean mass = ↓1.70% from pre- to post spaceflight mission.
Smith et al. 2012	45-47 yrs (Male)	N = 11 (iRED=8 and ARED=5)	To investigate the benefits of resistance training and nutrition in long-duration spaceflight on bone parameters.	160	Crew were scheduled 2.5 hours per day for exercise and this included time for setup and personal hygiene. Aerobic exercise was prescribed for cycle ergometer or treadmill 6 days per week for approximately 30 minutes. Detailed data on intensity and adherence are not available. Resistance exercise was prescribed 6 days per week and the lower body exercises included squats, heel raises, and deadlifts on both iRED and ARED. ARED allows the subject to vary the stance width, and accordingly the ARED prescription includes both normal and wide stance postures for squat and deadlift whereas iRED exercise was limited to a narrow stance.	Bone density, body mass composition.	Bone mineral density = ↓4% (iRED) and ↓1% (ARED), pelvis bone mineral density = ↓12% (iRED) and ↓2% (ARED). Total fat = ↑2% (iRED) and ↓12% (ARED), total lean = ↓1% (iRED) and ↑4% (ARED) from pre-to post spaceflight mission.
Trappe et al. 2009	45-47 yrs (Male)	N = 9	To document the exercise program used by crewmembers while aboard the ISS for 6 months and examine its effectiveness for preserving calf muscle characteristics.	180	Crewmembers were on the ISS, they had access to a treadmill (treadmill with vibration isolation system), two bicycle ergometers (cycle ergometer with vibration isolation system and a Velosiped, i.e., Russian bicycle exercise device), and an iRED. The crew members also had access to bungee cords, which they could use to provide resistance-type exercise for various muscle groups. The iRED is an elastomer-based resistance exercise device consisting of two canisters capable of producing up to 68 kg of force per canister. Additional bungee cords can also be attached to increase the load characteristics. resistance exercise at least 3 days/wk and several	Muscle volume and muscle strength	Gastrocnemius volume = ↓10%, soleuos = ↓15% and gastrocnemuus + soleus = ↓13% from pre- to revovery spacefligth mission. Muscle isokinetic 60 graus/sec = ↓24.21% and 180 graus/sec = ↓32.55% from pre- to revovery spacefligth mission.

conducting resistance training 5–6 days/wk. During the resistance training sessions, crewmembers averaged 3–6 sets of 12–20 repetitions for each leg exercise.

Note. ARED = Advanced Resistive Exercise Device; ISS = International Space Station; VO_2peak = Peak oxygen uptake; iRED = Interim Resistive Exercise Device; ESA = European Space Agency's; LDM = Long-Duration Missions; aBMD = Area Bone Mineral Density.
