# Supplementary material for: Disuse‐Induced Muscle Atrophy and Muscle Weakness From Hospitalization to Spaceflight: Exercise Succeeds in Prevention and Treatment—A Meta‐Analysis
Source: J Cachexia Sarcopenia Muscle. 2026 Apr 15;17(2):e70259. doi: 10.1002/jcsm.70259 (PMC13080877; doi:10.1002/jcsm.70259)
Supplement: Supplementary file 5 — Table S5: Studies characteristics of individuals recovering from disuse condition. [file JCSM-17-e70259-s008.pdf]

**Table S4.** Studies characteristics of individuals recovering from disuse condition.

| Author/Year | Age/Sex | Sample | Objective | Duration (days) | Exercise protocol | Measurement | Main outcome |
|---|---|---|---|---|---|---|---|
| Echeverria et al. 2020 | 77-87 yrs (Male/Female) | N = 36 (ST= 19 and LT= 17) | To compare the effects of two group-based multicomponent exercise interventions of different lengths in older adults after hospitalization. | 90 and 180 | Both groups were under twice a week, 40-70% of 1-RM, 2-3 sets of 8-10 reps (arm curl, leg flexion, hip extension, leg abduction, standing on tiptoes and heels, and chair stand). | SPPB, 8-m walking speed test, senior fitness test, 30-s chair-stand test and 8-ft TUGT. | SPPB = ↑ 1.9 kg (ST) and ↑ 2.3 kg (LT); 8-m test = ↑ 0.11 m/s (ST) and ↑ 0.12 m/s (LT); 30-s chair-stand = ↑ 2.9 (ST) and ↑ 2.8 (LT). |
| Hacker et al. 2017 | 60 yrs (Male/Female) | N = 67 (ST = 33 and UC = 34) | It was to determine the efficacy of an exercise intervention, strength training to enhance early recovery (STEER), on physical activity, fatigue, muscle strength, functional ability, and quality of life after hematopoietic stem cell transplantation. | 90 | ST consisted of 11 preselected exercises with concentric and eccentric muscle contractions as follows: (1) 8 exercises using elastic resistance bands (chest fly, biceps curl, triceps extension, shoulder shrug, shoulder upright row, shoulder raise, knee flexion, and knee extension) and (2) 3 exercises that used body weight as resistance (wall push-ups, squats, and bed sit-ups). Three times per week, 1-2 sets of maximum repetition, 13 pts of BORG scale 20-pts. | Handgrip, arm curl test, 8-ft TUGT and chair-stand test. | Handgrip = ↓ 2.94 (ST) and ↓ 3.83 (UC); arm curl test = ↑ 0.57 (ST) and ↓ 1.32 (UC); TUG = ↓ 0.13 (ST) and ↑ 2.25 (UC) and chair-stand test = ↔ (ST) and ↓ 1.03 (UC). |
| Rittweger et al. 2007 | 28-36 yrs (Male) | N = 24 (RT = 8 and Control = 16) | To test the efficacy of flywheel resistive exercise and pamidronate to counteract such losses. | 90 | It employed a flywheel resistance exercise regimen (i.e., four sets of seven maximal concentric and eccentric actions every third day) that has been effective in promoting quadriceps hypertrophy in both ambulatory and unloaded subjects in response to 5-week interventions. | Muscle power | Lower-body peak power = ↔ in RT group compared with Control group. |
| Suetta et al. 2008 | 60-86 yrs (Male/Female) | N = 36 (RT = 18 and Control = 18) | To compare different training regimes with respect to muscle strength, muscle fiber size, muscle architecture, and stair walking power in elderly postoperative patients. | 90 | Participants were randomized to either resistance training (3/wk 12 wk) or standard rehabilitation (Control group: 1 h/day 12 wk). | Muscle strength and size | Maximal dynamic muscle strength = + 30% in RT group compared with Control group. Muscle fiber area = type I (+ 17%), type IIa (+ 37%) and type IIx muscle fibers (+ 51%). |
| Timonen et al. 2002 | 75 yrs (Female) | N = 68 (TG = 34 and Control = 34) | To investigate the effects of an outpatient multicomponent training program including strength training on muscle strength, balance and walking speed in old, home-dwelling, frail | 254 | Subjects assigned to a training group started training usually 1 week after discharge. Training classes were given twice a week, for a 10-week period (20 sessions, 90min each), and the training sessions were supervised by two physiotherapists. The training phase lasted about 30 min. During the first four sessions, only low weights were used with 20–30 repetitions. Thereafter, the loads were gradually | Knee extension (%), hip abduction (%) and maximal walking (%). | Knee extension % (1 week) = ↑ 20.8 (TG) and ↑ 5.1 (Control); hip abduction % = ↑ 13.2 (TG) and ↑ 5.6 (Control); maximal walking % = ↑ 0.12 (TG) and ↓ 0.05 (Control). Knee extension % (3 months) = ↑ 21.3 (TG) and ↑ 6.7 (Control); hip abduction % = ↑ 12 (TG) and ↓ 4.5 (Control); maximal |

| | | | | | | |
|---|---|---|---|---|---|---|
| | | women recently discharged from a hospital ward, and to determine how long such effects may last. | | increased to the point where the participant could accomplish only 8–10 repetitions in two sets with the encouragement of the trainer. | | walking % = ↑ 0.11 (TG) and ↓ 0.09 (Control).<br>Knee extension % (9 month) = ↑ 7.9 (TG) and ↓ 3.2 (Control); hip abduction % = ↑ 9 (TG) and ↓ 11.8 (Control); maximal walking % = ↑ 0.05 (TG) and ↓ 0.09 (Control). |

**Note.** ST= short-term intervention; LT = long-term intervention; SPPB = Short Physical Performance Battery; 8-ft TUGT = 8-ft timed up-and-go test; UC = Usual Care; RT = Resistance Training.
