# Supplementary material for: Disuse‐Induced Muscle Atrophy and Muscle Weakness From Hospitalization to Spaceflight: Exercise Succeeds in Prevention and Treatment—A Meta‐Analysis
Source: J Cachexia Sarcopenia Muscle. 2026 Apr 15;17(2):e70259. doi: 10.1002/jcsm.70259 (PMC13080877; doi:10.1002/jcsm.70259)
Supplement: Supplementary file 7 — Data S2: Supporting information. [file JCSM-17-e70259-s007.pdf]

<u>Systematic Search</u>

Pubmed

((((((((((((((((((((((((((((((("acute hospitalization")) OR ("hospitalized individuals")) OR ("hospital-based care")) OR ("Inpatients")) AND ("bed rest")) OR ("bedridden individuals")) OR ("limb-immobilization")) OR ("immobilization")) OR ("Hind-limb")) AND ("spaceflight missions")) OR ("aerospace flight")) OR ("long-duration space mission")) OR ("international space station")) OR ("astronauts")) AND ("physical exercise")) OR ("aerobic training")) OR ("endurance training")) OR ("resistance training")) OR ("structured exercise training")) OR ("combined exercise training")) OR ("resistance exercise")) OR ("multicomponent exercise")) OR ("weight training")) OR ("interim resistive exercise device")) OR ("resistive device")) AND ("muscle mass")) OR ("lean body mass")) OR ("muscle size")) OR ("cross-sectional area")) OR ("muscle strength") OR ("muscular strength") OR ("muscle force") OR ("muscle power") OR ("dynamic muscle strength") OR ("handgrip strength") OR ("muscle contraction") OR ("maximum voluntary contraction") AND ("Randomized clinical trial")

Scopus

TITLE-ABS ("acute hospitalization" OR "hospitalized individuals" OR "hospital-based care" OR "Inpatients") AND ("Bed rest" OR "bedridden individuals" OR "limb-immobilization" OR "immobilization" OR "Hind-limb" OR "spaceflight missions" OR "aerospace flight" OR "long duration space mission" OR "international space station" OR "Astronauts") AND ("physical exercise" OR "aerobic training" OR "endurance training" OR "resistance training" OR "structured exercise training" OR "combined exercise training" OR "resistance exercise" OR "multicomponent exercise" OR "weight training" OR "interim resistive exercise device" OR "resistive device") AND ("muscle mass" OR "lean body mass" OR "muscle size" OR "cross sectional area" OR "muscle strength" OR "muscular strength" OR "muscle force" OR "muscle power" OR "dynamic muscle strength" OR "handgrip strength" OR "muscle contraction" OR "maximum voluntary contraction")

Web of Science

TI=("acute hospitalization" OR "hospitalized individuals" OR "hospital-based care" OR "Inpatients" OR "Bed rest" OR "bedridden individuals" OR "limb-immobilization" OR "immobilization" OR "Hind-limb" OR "spaceflight missions" OR "aerospace flight" OR "long duration space mission" OR "international space station" OR "Astronauts") AND TI=("physical exercise" OR "aerobic training" OR "endurance training" OR "resistance training" OR "structured exercise training" OR "combined exercise training" OR "resistance exercise" OR "multicomponent exercise" OR "weight training" OR "interim resistive exercise device" OR "resistive device") AND TI=("muscle mass" OR "lean body mass" OR "muscle size" OR "cross sectional area" OR "muscle strength" OR "muscular strength" OR "muscle force" OR "muscle power" OR "dynamic muscle strength" OR "handgrip strength" OR "muscle contraction" OR "maximum voluntary contraction")

SPORTDiscus full-text

TI ("acute hospitalization" OR "hospitalized individuals" OR "hospital-based care" OR "Inpatients") AND TI ("Bed rest" OR "bedridden individuals" OR "limb-immobilization" OR "immobilization" OR "Hind-limb" OR "spaceflight missions" OR "aerospace flight" OR "long duration space mission" OR "international space station" OR "Astronauts") AND TI ("physical exercise" OR "aerobic training" OR "endurance training" OR "resistance training" OR "structured exercise training" OR "combined exercise training" OR "resistance exercise" OR "multicomponent exercise" OR "weight training" OR "interim resistive exercise device" OR "resistive device") AND TI ("muscle mass" OR "lean body mass" OR "muscle size" OR "cross sectional area" OR "muscle strength" OR "muscular strength" OR "muscle force" OR "muscle power" OR "dynamic muscle strength" OR "handgrip strength" OR "muscle contraction" OR "maximum voluntary contraction")

CINAHL full-text

TI ("acute hospitalization" OR "hospitalized individuals" OR "hospital-based care" OR "Inpatients") AND TI ("Bed rest" OR "bedridden individuals" OR "limb-immobilization" OR "immobilization" OR "Hind-limb" OR "spaceflight missions" OR "aerospace flight" OR "long duration space mission" OR "international space station" OR "Astronauts") AND TI ("physical exercise" OR "aerobic training" OR "endurance training" OR "resistance training" OR "structured exercise training" OR "combined exercise training" OR "resistance exercise" OR "multicomponent exercise" OR "weight training" OR "interim resistive exercise device" OR "resistive device") AND TI ("muscle mass" OR "lean body mass" OR "muscle size" OR "cross sectional area" OR "muscle strength" OR "muscular strength" OR "muscle force" OR "muscle power" OR "dynamic muscle strength" OR "handgrip strength" OR "muscle contraction" OR "maximum voluntary contraction")
