# Supplementary material for: Disuse‐Induced Muscle Atrophy and Muscle Weakness From Hospitalization to Spaceflight: Exercise Succeeds in Prevention and Treatment—A Meta‐Analysis
Source: J Cachexia Sarcopenia Muscle. 2026 Apr 15;17(2):e70259. doi: 10.1002/jcsm.70259 (PMC13080877; doi:10.1002/jcsm.70259)
Supplement: Supplementary file 8 — Data S3: Supporting information. [file JCSM-17-e70259-s005.pdf]

Full-text excluded

Author/year	Title	Reason
Steele J et al., 2019	Comparisons of Resistance Training and "Cardio" Exercise Modalities as Countermeasures to Microgravity-Induced Physical Deconditioning: New Perspectives and Lessons Learned From Terrestrial Studies.	Different comparators
Burkhart K et al., 2019	Negative Effects of Long-duration Spaceflight on Paraspinal Muscle Morphology.	Different comparators
Zemková E and Oddsson L, 2016	Effects of Stable and Unstable Resistance Training in an Altered-G Environment on Muscle Power.	Incomplete endpoints
Schneider S et al., 2015	Feasibility of monitoring muscle health in microgravity environments using Myoton technology.	Different comparators
Matsuse H et al., 2006	Muscle training by means of combined electrical stimulation and volitional contraction.	Incomplete endpoints
Tesch PA et al., 2004	Hypertrophy of chronically unloaded muscle subjected to resistance exercise.	Different comparators
Aarden JJ et al., 2021	Longitudinal Changes in Muscle Mass, Muscle Strength, and Physical Performance in Acutely Hospitalized Older Adults	Participants under chronic treatment
Sáez de Asteasu ML et al., 2019	Assessing the impact of physical exercise on cognitive function in older medical patients during acute hospitalization: Secondary analysis of a randomized trial	Incomplete endpoints
Arentson-Lantz E et al., 2019	2,000 steps/day does not fully protect skeletal muscle health in older adults during bed rest	Wrong outcome
Åhlund K et al., 2018	Physical Performance Impairments and Limitations among Hospitalized Frail Older Adults	Wrong outcome
Jones SE et al., 2018	Pulmonary rehabilitation in patients with an acute exacerbation of chronic obstructive pulmonary disease	Participants under chronic treatment
Sarmento LA et al. 2017	Effect of conventional physical therapy and Pilates in functionality, respiratory muscle strength and ability to exercise in hospitalized chronic renal patients: A randomized controlled trial	Participants under chronic treatment
Martínez-Velilla N et al., 2015	Functional and cognitive impairment prevention through early physical activity for geriatric hospitalized patients: Study protocol for a randomized controlled trial	Wrong outcome
Wakabayashi H and Sakuma K et al., 2014	Rehabilitation nutrition for sarcopenia with disability: a combination of both rehabilitation and nutrition care management	Different comparators
Dennis RA et al., 2012	Changes in activities of daily living, nutrient intake, and systemic inflammation in elderly adults receiving recuperative care	Participants under chronic treatment
Ohira Y et al., 2008	Comparison of inpatients' physical activity between pre and post discharge to home	Incomplete endpoints

Crevenna R et al., 2004	Long-term Transcutaneous Neuromuscular Electrical Stimulation in Patients with Bipolar Sensing Implantable Cardioverter Defibrillators: A Pilot Safety Study	Wrong outcome
Hauer K et al., 2002	Intensive physical training in geriatric patients after severe falls and hip surgery	Participants under chronic treatment
Kasper CE 2001	Functional loss: Aging muscle during rehabilitation	Different comparators
Macias Brandon R et al., 2020	Association of Long-Duration Spaceflight With Anterior and Posterior Ocular Structure Changes in Astronauts and Their Recovery	Wrong outcome
Christensen PA et al., 2008	Changes in maximum muscle strength and rapid muscle force characteristics after long-term special support and reconnaissance missions: a preliminary report	Incomplete endpoints
Hurst IV et al., 2015	Concept of Operations Evaluation for Using Remote - Guidance Ultrasound for Exploration Spaceflight	Wrong outcome
Roberts DR et al., 2017	Effects of Spaceflight on Astronaut Brain Structure as Indicated on MRI	Incomplete endpoints
Britt TW et al., 2017	Enhancing the Meaningfulness of Work for Astronauts on Long Duration Space Exploration Missions	Wrong outcome
Laughlin MS et al., 2015	Functional Fitness Testing Results Following Long-Duration ISS Missions	Different comparators
Blottner D et al., 2006	Human skeletal muscle structure and function preserved by vibration muscle exercise following 55 days of bed rest	Incomplete endpoints
Manzey D et al., 2000	Impairments of manual tracking performance during spaceflight: more converging evidence from a 20-day space mission	Incomplete endpoints
Piquet V et al., 2021	Do Patients With COVID-19 Benefit from Rehabilitation? Functional Outcomes of the First 100 Patients in a COVID-19 Rehabilitation Unit	Different comparators
Eldawati et al. 2020	The effectiveness pre-operative exercise of muscle strength for early ambulation on lower limb fracture with measurement tool – The Modified Iowa Level of Assistance Scale (MILAS) – in hospital inpatients	Incomplete endpoints
Fukushima T et al., 2020	Factors associated with muscle function in patients with hematologic malignancies undergoing chemotherapy	Participants under chronic treatment
Svinøy OE et al., 2019	Better before-better after: Efficacy of prehabilitation for older patients with osteoarthritis awaiting total hip replacement - A randomised controlled trial in South-Eastern Norway	Different comparators
Duregon F et al., 2019	Exercise prescription and tailored physical activity intervention in onco-hematology inpatients, a personalized bedside approach to improve clinical best practice	Participants under chronic treatment
Åhlund K et al., 2018	Physical Performance Impairments and Limitations among Hospitalized Frail Older Adults	Incomplete endpoints

Chang DG et al., 2016	Lumbar Spine Paraspinal Muscle and Intervertebral Disc Height Changes in Astronauts After Long-Duration Spaceflight on the International Space Station	Incomplete endpoints
Moore Jr et al., 2014	Peak exercise oxygen uptake during and following long-duration spaceflight	Incomplete endpoints
Kukoba TB et al., 2019	Preventive Efficiency of Resistive Exercises for the Bone System of Cosmonauts in Repeated Long-Duration Space Missions	Incomplete endpoints
Hackney KJ et al., 2015	The Astronaut-Athlete: Optimizing Human Performance in Space	Incomplete endpoints
Gaffney C et al., 2017	The effect of long-term confinement and the efficacy of exercise countermeasures on muscle strength during a simulated mission to Mars: data from the Mars500 study	Incomplete endpoints
Hoffmann U et al., 2016	$\dot{V}O2$ and HR kinetics before and after International Space Station missions	Wrong outcome
Palmieri-Smith RM et al., 2022	Functional Resistance Training Improves Thigh Muscle Strength after ACL Reconstruction: A Randomized Clinical Trial.	Incomplete endpoints
Al Anazi A et al., 2022	Handgrip Strength Exercises Modulate Shoulder Pain, Function, and Strength of Rotator Cuff Muscles of Patients with Primary Subacromial Impingement Syndrome	Participants under chronic treatment
Min J et al., 2024	Early Implementation of Exercise to Facilitate Recovery After Breast Cancer Surgery: A Randomized Clinical Trial	Participants under chronic treatment
Fossat G et al., 2018	Effect of In-Bed Leg Cycling and Electrical Stimulation of the Quadriceps on Global Muscle Strength in Critically Ill Adults: A Randomized Clinical Trial	Wrong outcome
de Paula MAS et al., 2024	Effect of a structured early mobilization protocol on the level of mobilization and muscle strength in critical care patients: A randomized clinical trial	Wrong outcome
Sáez de Asteasu ML et al., 2024	Dose-Response Relationship Between Exercise Duration and Enhanced Function and Cognition in Acutely Hospitalized Older Adults: A Secondary Analysis of a Randomized Clinical Trial	Incomplete endpoints
Ramírez-Vélez R et al., 2021	Handgrip Strength as a Complementary Test for Mobility Limitations Assessment in Acutely Hospitalized Oldest Old	Incomplete endpoints
Flores J et al., 2023	Effects of an early rehabilitation program for adult cystic fibrosis patients during hospitalization: a randomized clinical trial	Participants under chronic treatment
Bays-Moneo AB et al., 2023	Cost-Consequences Analysis Following Different Exercise Interventions in Institutionalized Oldest Old: A Pilot Study of a Randomized Clinical Trial	Participants under chronic treatment

Sáez de Asteasu ML et al., 2019	Physical Exercise Improves Function in Acutely Hospitalized Older Patients: Secondary Analysis of a Randomized Clinical Trial	Wrong outcome
Krištof Mirt P et al., 2022	Effects of early home-based strength and sensory-motor training after total hip arthroplasty: study protocol for a multicenter randomized controlled trial	Incomplete endpoints
Cook SB et al. 2010	Skeletal muscle adaptations following blood flow-restricted training during 30 days of muscular unloading	Incomplete endpoints
Gatty et al., 2020	Effectiveness of structured early mobilization protocol on mobility status of patients in medical intensive care unit	Wrong outcome
Zätterström R et al., 2000	Rehabilitation following acute anterior cruciate ligament injuries--a 12-month follow-up of a randomized clinical trial	Different comparators
Machado ADS et al., 2017	Effects that passive cycling exercise have on muscle strength, duration of mechanical ventilation, and length of hospital stay in critically ill patients: a randomized clinical trial	Different comparators
Pandey A et al., 2023	Frailty and Effects of a Multidomain Physical Rehabilitation Intervention Among Older Patients Hospitalized for Acute Heart Failure: A Secondary Analysis of a Randomized Clinical Trial	Different comparators
Ahmad F et al., 2023	De-frailing intervention for hospitalized cardiovascular patients in the TARGET-EFT randomized clinical trial	Wrong outcome
Hajj-Boutros G et al., 2023	Impact of 14 Days of Bed Rest in Older Adults and an Exercise Countermeasure on Body Composition, Muscle Strength, and Cardiovascular Function: Canadian Space Agency Standard Measures.	Different comparators
Krainski F et al., 2014	The effect of rowing ergometry and resistive exercise on skeletal muscle structure and function during bed rest.	Different comparators
Fernandez-Gonzalo R et al., 2014	Flywheel resistance exercise to maintain muscle oxidative potential during unloading	Incomplete endpoints
Bamman MM et al., 1997	Resistance exercise prevents plantar flexor deconditioning during bed rest	Incomplete endpoints
Tesch PA et al., 2013	Cardiovascular responses to rowing on a novel ergometer designed for both resistance and aerobic training in space	Incomplete endpoints
Kramer A et al. 2017	How to prevent the detrimental effects of two months of bed-rest on muscle, bone and cardiovascular system: an RCT	Wrong outcome
Kramer A et al. 2021	Daily 30-min exposure to artificial gravity during 60 days of bed rest does not maintain aerobic exercise capacity but mitigates some deteriorations of muscle function: results from the AGBRESA RCT	Incomplete endpoints
Wang H et al. 2012	Resistive vibration exercise retards bone loss in weight-bearing skeletons during 60 days bed rest	Different comparators

Coupé M et al., 2011	Low-magnitude whole body vibration with resistive exercise as a countermeasure against cardiovascular deconditioning after 60 days of head-down bed rest	Different comparators
Cao P et al. 2005	Exercise within lower body negative pressure partially counteracts lumbar spine deconditioning associated with 28-day bed rest	Different comparators
Yang C et al., 2014	Effects of 60-day head-down bed rest on osteocalcin, glycolipid metabolism and their association with or without resistance training	Wrong outcome
Siebens H et al., 2000	A randomized controlled trial of exercise to improve outcomes of acute hospitalization in older adults	Different comparators
Kuzmik A et al., 2023	Gender, Pain, and Function Associated With Physical Activity After Hospitalization in Persons Living With Dementia	Different comparators
Berney S et al., 2012	Safety and feasibility of an exercise prescription approach to rehabilitation across the continuum of care for survivors of critical illness	Wrong outcome
Martínez-Velilla N et al., 2021	Recovery of the Decline in Activities of Daily Living After Hospitalization Through an Individualized Exercise Program: Secondary Analysis of a Randomized Clinical Trial	Incomplete endpoints
Martínez-Velilla N et al., 2022	Effect of an Exercise Intervention on Functional Decline in Very Old Patients During Acute Hospitalizations: Results of a Multicenter, Randomized Clinical Trial	Wrong outcome
Schweickert WD et al., 2009	Early physical and occupational therapy in mechanically ventilated, critically ill patients: a randomised controlled trial	Different comparators
Koppelmans V et al., 2018	Exercise effects on bed rest-induced brain changes	Wrong outcome
Hoff P et al., 2015	Effects of 60-day bed rest with and without exercise on cellular and humoral immunological parameters	Wrong outcome
Kenny HC et al., 2017	Bed rest and resistive vibration exercise unveil novel links between skeletal muscle mitochondrial function and insulin resistance	Different comparators
Miokovic T et al., 2014	Muscle atrophy, pain, and damage in bed rest reduced by resistive (vibration) exercise	Incomplete endpoints
Mutin-Carnino M et al., 2024	Effect of muscle unloading, reloading and exercise on inflammation during a head-down bed rest	Incomplete endpoints
Dillon EL et al., 2018	Efficacy of Testosterone plus NASA Exercise Countermeasures during Head-Down Bed Rest	Different comparators
van Duijnhoven NT et al., 2008	The effect of bed rest and an exercise countermeasure on leg venous function	Wrong outcome
Belavý DL et al., 2012	Resistive vibration exercise during bed-rest reduces motor control changes in the lumbo-pelvic musculature	Different comparators
Gast U et al. 2012	Short-duration resistive exercise sustains neuromuscular function after bed rest	Different comparators

Mulder E et al., 2014	Study protocol, implementation, and verification of a short versatile upright exercise regime during 5 days of bed rest	Incomplete endpoints
Bergouignan A et al., 2010	Regulation of energy balance during long-term physical inactivity induced by bed rest with and without exercise training	Incomplete endpoints
Waha JE et al., 2015	Effects of Exercise and Nutrition on the Coagulation System During Bedrest Immobilization	Wrong outcome
Lozano-Vicario L et al. 2024	Effects of Exercise Intervention for the Management of Delirium in Hospitalized Older Adults: A Randomized Clinical Trial	Different comparators
Martínez-Velilla N et al., 2021	Effects of a Tailored Exercise Intervention in Acutely Hospitalized Oldest Old Diabetic Adults: An Ancillary Analysis	Different comparators
Falavigna LF et al., 2014	Effects of electrical muscle stimulation early in the quadriceps and tibialis anterior muscle of critically ill patients	Different comparators
Frazão M et al., 2025	Recumbent FES-Cycling Exercise Improves Muscle Performance and Ambulation Capacity in Hospitalized Patients: A Randomized Controlled Trial	Different comparators
Schott N et al., 2014	Preventing functional loss during immobilization after osteoporotic wrist fractures in elderly patients: a randomized clinical trial	Different comparators
Deer RR et al., 2019	A Phase I Randomized Clinical Trial of Evidence-Based, Pragmatic Interventions to Improve Functional Recovery After Hospitalization in Geriatric Patients	Different comparators
Deer RR et al., 2018	A Randomized Controlled Pilot Trial of Interventions to Improve Functional Recovery After Hospitalization in Older Adults: Feasibility and Adherence	Different comparators
Hargens AR et al., 2016	Long-duration bed rest as an analog to microgravity	Incomplete endpoints
Trappe TA et al., 2023	Microgravity-induced skeletal muscle atrophy in women and men: implications for long-duration spaceflights to the Moon and Mars	Incomplete endpoints
Lee SM et al., 2014	WISE-2005: Countermeasures to prevent muscle deconditioning during bed rest in women	Incomplete endpoints
Guinet P et al., 2020	MNX (Medium Duration Nutrition and Resistance-Vibration Exercise) Bed-Rest: Effect of Resistance Vibration Exercise Alone or Combined With Whey Protein Supplementation on Cardiovascular System in 21-Day Head-Down Bed Rest	Different comparators
Kermorgant M et al., 2021	Effects of Resistance Exercise with or without Whey Protein Supplementation on Ocular Changes after a 21-Day Head-Down Bed Rest	Wrong outcome
Kermorgant M et al., 2019	Effects of Resistance Exercise and Nutritional Supplementation on Dynamic Cerebral Autoregulation in Head-Down Bed Rest	Wrong outcome

Carrick-Ranson G et al., 2013	The effect of exercise training on left ventricular relaxation and diastolic suction at rest and during orthostatic stress after bed rest	Wrong outcome
Trappe TA et al., 2024	NASA SPRINT exercise program efficacy for vastus lateralis and soleus skeletal muscle health during 70 days of simulated microgravity	Different comparators
Skiles CM et al., 2025	Myonuclear and satellite cell content of the vastus lateralis and soleus with 70 days of simulated microgravity and the NASA SPRINT exercise program	Different comparators
Gallagher P et al., 2005	Effects of 84-days of bedrest and resistance training on single muscle fibre myosin heavy chain distribution in human vastus lateralis and soleus muscles	Different comparators
Trappe TA et al., 2007	Influence of concurrent exercise or nutrition countermeasures on thigh and calf muscle size and function during 60 days of bed rest in women	Different comparators
Alley DE et al., 2010	Hospitalization and change in body composition and strength in a population-based cohort of older persons	Different comparators
Taya M et al., 2018	High-intensity aerobic interval training can lead to improvement in skeletal muscle power among in-hospital patients with advanced heart failure	Incomplete endpoints
Voss AC et al., 2024	Exercise microdosing for skeletal muscle health applications to spaceflight	Incomplete endpoints
Chang CL et al., 2025	Inspiratory muscle training and aerobic exercise for respiratory muscle strength in myasthenia gravis post-hospitalization- a randomized controlled trial	Incomplete endpoints
Dreiner M et al., 2025	Immobilization by 21-days of bed rest causes changes in biomarkers of cartilage homeostasis in healthy individuals	Wrong outcome
Deutz NE et al., 2016	Readmission and mortality in malnourished, older, hospitalized adults treated with a specialized oral nutritional supplement: A randomized clinical trial	Different comparators
Heels-Ansdell D et al., 2024	Early In-Bed Cycle Ergometry With Critically Ill, Mechanically Ventilated Patients: Statistical Analysis Plan for CYCLE (Critical Care Cycling to Improve Lower Extremity Strength), an International, Multicenter, Randomized Clinical Trial	Different comparators
Garbin AJ et al., 2024	Improving Function in Older Adults With Hospital-Associated Deconditioning: Lessons Learned Comparing a Randomized Controlled Trial to Real World Practice	Different comparators
Garbin AJ et al., 2024	Progressive Multicomponent Intervention for Older Adults in Home Health Settings Following Hospitalization: Randomized Clinical Trial	Incomplete endpoints
Philippot A et al., 2022	Impact of physical exercise on depression and anxiety in adolescent inpatients: A randomized controlled trial	Ineligible participant

Kitzman DW et al., 2021	Physical Rehabilitation for Older Patients Hospitalized for Heart Failure	Wrong outcome
Tor-Roca A et al., 2023	Adherence to Mediterranean Diet and Response to an Exercise Program to Prevent Hospitalization-Associated Disability in Older Adults: A Secondary Analysis from a Randomized Controlled Trial	Wrong outcome
McCullagh R et al., 2020	Augmented exercise in hospital improves physical performance and reduces negative post hospitalization events: a randomized controlled trial	Different comparators
Nakanishi N et al., 2020	Upper limb muscle atrophy associated with in-hospital mortality and physical function impairments in mechanically ventilated critically ill adults: a two-center prospective observational study	Wrong outcome
Wernhart S et al., 2021	The Feasibility of High-Intensity Interval Training in Patients with Intensive Care Unit-Acquired Weakness Syndrome Following Long-Term Invasive Ventilation	Wrong outcome
English KL et al., 2015	Isokinetic Strength Changes Following Long-Duration Spaceflight on the ISS	Incomplete endpoints
English KL et al., 2019	Exercise Countermeasures to Neuromuscular Deconditioning in Spaceflight	Incomplete endpoints
McCullagh R et al., 2016	A study protocol of a randomised controlled trial to measure the effects of an augmented prescribed exercise programme (APEP) for frail older medical patients in the acute setting	Wrong outcome
Fregly BJ et al., 2015	Computational Prediction of Muscle Moments During ARED Squat Exercise on the International Space Station	Different comparators
Blottner D et al., 2006	Human skeletal muscle structure and function preserved by vibration muscle exercise following 55 days of bed rest	Different comparators
Moriggi M et al., 2010	Long term bed rest with and without vibration exercise countermeasures: effects on human muscle protein dysregulation	Wrong outcome
Lambertz D et al., 2001	Effects of long-term spaceflight on mechanical properties of muscles in humans	Different comparators
Koryak YA 2020	Isokinetic Force and Work Capacity After Long-Duration Space Station Mir and Short-Term International Space Station Missions	Different comparators
Gopalakrishnan R et al., 2010	Muscle volume, strength, endurance, and exercise loads during 6-month missions in space	Incomplete endpoints
Lambertz D et al., 2003	Influence of long-term spaceflight on neuromechanical properties of muscles in humans	Wrong outcome
Iellamo F et al., 2006	Muscle metaboreflex contribution to cardiovascular regulation during dynamic exercise in microgravity: insights from mission STS-107 of the space shuttle Columbia	Wrong outcome
Yang Y et al., 2007	Space cycle: a human-powered centrifuge that can be used for hypergravity resistance training	Different comparators

Agha NH et al., 2020	Exercise as a countermeasure for latent viral reactivation during long duration space flight	Different comparators
Lee SM et al., 2007	Supine LBNP exercise maintains exercise capacity in male twins during 30-d bed rest	Different comparators
Smith SM et al., 2003	Evaluation of treadmill exercise in a lower body negative pressure chamber as a countermeasure for weightlessness-induced bone loss: a bed rest study with identical twins	Incomplete endpoints
Lee SM et al., 2009	LBNP exercise protects aerobic capacity and sprint speed of female twins during 30 days of bed rest	Different comparators
Naseri C et al., 2022	Factors Affecting Engagement of Older Adults in Exercise Following Hospitalization	Different comparators
Rodriguez-Lopez C et al., 2024	Exercise Intervention and Hospital-Associated Disability: A Nonrandomized Controlled Clinical Trial	Different comparators
Ortiz-Alonso J et al., 2020	Effect of a Simple Exercise Program on Hospitalization-Associated Disability in Older Patients: A Randomized Controlled Trial	Different comparators
Knaut C et al., 2017	Assessment of Aerobic Exercise Adverse Effects during COPD Exacerbation Hospitalization	Patients under chronic treatment
Farag I et al., 2015	Cost-effectiveness of a Home-Exercise Program Among Older People After Hospitalization	Different comparators
Karapınar M et al., 2022	Effectiveness of supervised and functional level-based exercise program in elderly inpatients: Randomised controlled trial	Different comparators
Paneroni M et al., 2024	Home-based exercise program for people with residual disability following hospitalization for COVID-19: Randomized control trial	Different comparators
Hackney KJ et al., 2012	Blood flow-restricted exercise in space	Wrong outcome
Kouzaki M et al., 2007	Effects of 20-day bed rest with and without strength training on postural sway during quiet standing	Different comparators
Bleeker MW et al., 2005	Vascular adaptation to deconditioning and the effect of an exercise countermeasure: results of the Berlin Bed Rest study	Wrong outcome
Cromwell RL et al., 2018	Overview of the NASA 70-day Bed Rest Study	Wrong outcome
Deer RR et al., 2016	Identifying effective and feasible interventions to accelerate functional recovery from hospitalization in older adults: A randomized controlled pilot trial	Wrong outcome
Turunen K et al., 2017	A tailored counseling and home-based rehabilitation program to increase physical activity and improve mobility among community-dwelling older people after hospitalization: protocol of a randomized controlled trial	Wrong outcome
Valenzuela PL et al., 2025	Exercise effects on intrinsic capacity in acutely hospitalised older adults: a pooled analysis of two randomised controlled trials	Different comparators
Kramer A et al., 2017	High-Intensity Jump Training Is Tolerated during 60 Days of Bed Rest and Is Very Effective in Preserving	Different comparators

	Leg Power and Lean Body Mass: An Overview of the Cologne RSL Study	
Brooks NE et al., 2010	Effects of resistance exercise combined with essential amino acid supplementation and energy deficit on markers of skeletal muscle atrophy and regeneration during bed rest and active recovery	Different comparators
Stevens L et al., 2013	Potential regulation of human muscle plasticity by MLC2 post-translational modifications during bed rest and countermeasures	Wrong outcome
Belavý DL et al., 2008	Resistive simulated weightbearing exercise with whole body vibration reduces lumbar spine deconditioning in bed-rest	Different comparators
DeBusk RF et al., 1983	Exercise conditioning in middle-aged men after 10 days of bed rest	Incomplete endpoints
Sáez de Asteasu ML et al., 2021	Cognitive Function Improvements Mediate Exercise Intervention Effects on Physical Performance in Acutely Hospitalized Older Adults	Different comparators
Rittweger J and Felsenberg D 2009	Recovery of muscle atrophy and bone loss from 90 days bed rest: results from a one-year follow-up	Wrong outcome
Sivarajan ES et al., 1981	In-hospital exercise after myocardial infarction does not improve treadmill performance	Different comparators
Zwart SR et al., 2007	Lower body negative pressure treadmill exercise as a countermeasure for bed rest-induced bone loss in female identical twins	Wrong outcome
Mulder ER et al., 2006	Strength, size and activation of knee extensors followed during 8 weeks of horizontal bed rest and the influence of a countermeasure	Different comparators
Dilani Mendis M et al.,	Effect of prolonged bed rest on the anterior hip muscles	Wrong outcome
Mallery LH et al., 2003	The feasibility of performing resistance exercise with acutely ill hospitalized older adults	Wrong outcome
Choong K et al., 2017	Early Exercise in Critically Ill Youth and Children, a Preliminary Evaluation: The wEECYCLE Pilot Trial	Ineligible participant
Núñez-Cortés R et al., 2024	Acute Effects of In-Hospital Resistance Training on Clinical Outcomes in Patients Undergoing Total Knee Arthroplasty: A Randomized Controlled Trial	Different comparators
Liao LY et al., 2015	Efficacy of a respiratory rehabilitation exercise training package in hospitalized elderly patients with acute exacerbation of COPD: a randomized control trial	Patients under chronic treatment
Belin de Chantemèle E et al., 2004	Calf venous volume during stand-test after a 90-day bed-rest study with or without exercise countermeasure	Wrong outcome
Brooks N et al., 2008	Resistance training and timed essential amino acids protect against the loss of muscle mass and strength during 28 days of bed rest and energy deficit	Different comparators
Hides JA et al., 2011	The effects of rehabilitation on the muscles of the trunk following prolonged bed rest	Different comparators

Buehring B et al., 2011	Changes in lower extremity muscle function after 56 days of bed rest	Different comparators
Belavý DL et al., 2009	Differential atrophy of the lower-limb musculature during prolonged bed-rest	Wrong outcome
Shibata S et al., 2010	Supine cycling plus volume loading prevent cardiovascular deconditioning during bed rest	Wrong outcome
Naseri C et al., 2019	Evaluation of Tailored Falls Education on Older Adults' Behavior Following Hospitalization	Different comparators
Shinohara M et al., 2003	Strength training counteracts motor performance losses during bed rest	Incomplete endpoints
Sáez de Asteasu ML et al., 2024	Biological sex as a tailoring variable for exercise prescription in hospitalized older adults	Different comparators
Sandberg L et al., 2024	Feasibility of the "Preventing functional decline in acutely hospitalized older patients (PREV_FUNC)" study-A three-armed randomized controlled pilot trial	Different comparators
Mudge AM et al., 2018	Addition of Supervised Exercise Training to a Post-Hospital Disease Management Program for Patients Recently Hospitalized With Acute Heart Failure: The EJECTION-HF Randomized Phase 4 Trial	Participants under chronic treatment
Sherrington C et al., 2014	A post-hospital home exercise program improved mobility but increased falls in older people: a randomised controlled trial	Different comparators
Koifman E et al., 2014	Multidisciplinary rehabilitation program in recently hospitalized patients with heart failure and preserved ejection fraction: rationale and design of a randomized controlled trial	Different comparators
Zachwieja JJ et al., 1999	Testosterone administration preserves protein balance but not muscle strength during 28 days of bed rest	Different comparators
Belavý DL et al., 2011	Estimation of changes in volume of individual lower-limb muscles using magnetic resonance imaging (during bed-rest)	Different comparators
Raymond MJ et al., 2017	The effects of a high-intensity functional exercise group on clinical outcomes in hospitalised older adults: an assessor-blinded, randomised-controlled trial	Wrong outcome
Mudge AM et al., 2017	CHERISH (collaboration for hospitalised elders reducing the impact of stays in hospital): protocol for a multi-site improvement program to reduce geriatric syndromes in older inpatients	Wrong outcome
Chen J et al., 2021	The effect of in-hospital physiotherapy on handgrip strength and physical activity levels after cardiac valve surgery: a randomized controlled trial	Different comparators
Oikawa SY et al., 2018	A randomized controlled trial of the impact of protein supplementation on leg lean mass and integrated muscle protein synthesis during inactivity and energy restriction in older persons	Different comparators
Gill TM et al., 2017	The effect of intervening hospitalizations on the benefit of structured physical activity in promoting	Wrong outcome

	independent mobility among community-living older persons: secondary analysis of a randomized controlled trial	
Chantler I et al., 2006	Muscular strength changes in hospitalized anorexic patients after an eight week resistance training program	Different comparators
Eren B et al., 2020	Modified constraint-induced movement therapy during hospitalization in children with perinatal brachial plexus palsy: A randomized controlled trial	Ineligible participant
Mendes RG et al., 2010	Short-term supervised inpatient physiotherapy exercise protocol improves cardiac autonomic function after coronary artery bypass graft surgery--a randomised controlled trial	Different comparators
Arbeille P et al., 2008	Insufficient flow reduction during LBNP in both splanchnic and lower limb areas is associated with orthostatic intolerance after bedrest	Wrong outcome
Wu TT et al., 2024	Resistance training combined with β-hydroxy β-methylbutyrate for patients with critical illness: A four-arm, mixed-methods, feasibility randomised controlled trial	Different comparators
Marsh AP et al., 2016	Hospitalizations During a Physical Activity Intervention in Older Adults at Risk of Mobility Disability: Analyses from the Lifestyle Interventions and Independence for Elders Randomized Clinical Trial	Wrong outcome
Beelen J et al., 2017	A 12-week intervention with protein-enriched foods and drinks improved protein intake but not physical performance of older patients during the first 6 months after hospital release: a randomised controlled trial	Different comparators
Benzo et al., 2015	Implementation of physical activity programs after COPD hospitalizations: Lessons from a randomized study	Wrong outcome
Haennel et al., 1999	Effect of hydraulic circuit training following coronary artery bypass surgery	Participants under chronic treatment
Chen CY et al., 2015	Is rehabilitation intervention during hospitalization enough for functional improvements in patients undergoing lumbar decompression surgery? A prospective randomized controlled study	Different comparators
Checa-López M et al. 2024	Effectiveness of a randomized intervention by a geriatric team in frail hospital inpatients in non-geriatric settings: FRAILCLINIC project	Different comparators
Stegemann J et al., 1997	Exercise capacity during and after spaceflight	Different comparators
Convertino VA and Sandler H 1995	Exercise countermeasures for spaceflight	Different comparators
Hughes L et al., 2022	Optimization of Exercise Countermeasures to Spaceflight Using Blood Flow Restriction	Different comparators

Greene KA et al., 2022	Change in Lumbar Muscle Size and Composition on MRI with Long-Duration Spaceflight	Wrong outcome
Owerkowicz T et al., 2016	Exercise Responses to Gravity-Independent Flywheel Aerobic and Resistance Training	Different comparators
Cotter JA et al., 2015	Concurrent exercise on a gravity-independent device during simulated microgravity	Different comparators
Schulze K et al., 2002	Resistance training preserves skeletal muscle function during unloading in humans	Different comparators
Haus JM et al., 2007	Contractile and connective tissue protein content of human skeletal muscle: effects of 35 and 90 days of simulated microgravity and exercise countermeasures	Incomplete endpoints
Ploutz-Snyder LL et al., 2014	Integrated resistance and aerobic exercise protects fitness during bed rest	Different comparators
Carrithers JA et al., 2007	Concurrent exercise and muscle protein synthesis: implications for exercise countermeasures in space	Wrong outcome
Scott JPR et al., 2023	Effects of body size and countermeasure exercise on estimates of life support resources during all-female crewed exploration missions	Wrong outcome
Fränzel K et al., 2024	Square-stepping exercise in older inpatients in early geriatric rehabilitation. A randomized controlled pilot study	Different comparators
Chen MS et al., 2015	Aerobic and resistance exercise training program intervention for enhancing gait function in elderly and chronically ill Taiwanese patients	Wrong outcome
Sitjà-Rabert M 2015	Effects of a whole body vibration (WBV) exercise intervention for institutionalized older people: a randomized, multicentre, parallel, clinical trial	Different comparators
Chen MS et al., 2014	Resistance training exercise program for intervention to enhance gait function in elderly chronically ill patients: multivariate multiscale entropy for center of pressure signal analysis	Different comparators
McCall GE et al., 1999	Spaceflight suppresses exercise-induced release of bioassayable growth hormone	Different comparators
Berg HE et al., 1998	Force and power characteristics of a resistive exercise device for use in space	Incomplete endpoints
Zanini M et al., 2019	Effects of Different Rehabilitation Protocols in Inpatient Cardiac Rehabilitation After Coronary Artery Bypass Graft Surgery: A RANDOMIZED CLINICAL TRIAL	Different comparators
Tamulevičiūtė-Prascienė E et al., 2021	The impact of additional resistance and balance training in exercise-based cardiac rehabilitation in older patients after valve surgery or intervention: randomized control trial	Different comparators
Wolf F et al. 2023	Multimodal agility-based exercise training (MAT) versus strength and endurance training (SET) to improve multiple sclerosis-related fatigue and fatigability during inpatient rehabilitation: a randomized controlled pilot and feasibility study	Wrong outcome

Lee PY et al., 2020	Effects of Trunk Exercise on Unstable Surfaces in Persons with Stroke: A Randomized Controlled Trial	Different comparators
van de Port IG et al., 2012	Effects of circuit training as alternative to usual physiotherapy after stroke: randomised controlled trial	Different comparators
Fu Q et al., 2002	Cardiovascular and sympathetic neural responses to handgrip and cold pressor stimuli in humans before, during and after spaceflight	Wrong outcome
Pancera S et al., 2024	Effects of Combined Endurance and Resistance Eccentric Training on Muscle Function and Functional Performance in Patients With Chronic Obstructive Pulmonary Disease: Randomized Controlled Trial	Different comparators
Lau CW et al., 2023	Effect on muscle strength after blood flow restriction resistance exercise in early in-patient rehabilitation of post-chronic obstructive pulmonary disease acute exacerbation, a single blinded, randomized controlled study	Different comparators
Irimia JM et al., 2017	Metabolic adaptations in skeletal muscle after 84 days of bed rest with and without concurrent flywheel resistance exercise	Wrong outcome
Beigienė A et al., 2021	Frailty and Different Exercise Interventions to Improve Gait Speed in Older Adults after Acute Coronary Syndrome	Different comparators
Zong M et al., 2023	Effects of whey protein complex combined with low-intensity exercise in elderly inpatients with COPD at a stable stage	Different comparators
Groehs RV et al., 2016	Muscle electrical stimulation improves neurovascular control and exercise tolerance in hospitalised advanced heart failure patients	Wrong outcome
Greenleaf JE et al., 2006	Submaximal exercise VO2 and Qc during 30-day 6 degrees head-down bed rest with isotonic and isokinetic exercise training	Different comparators
Tang CY et al., 2012	Early rehabilitation exercise program for inpatients during an acute exacerbation of chronic obstructive pulmonary disease: a randomized controlled trial	Different comparators
Sunde S et al., 2020	Effects of a multicomponent high intensity exercise program on physical function and health-related quality of life in older adults with or at risk of mobility disability after discharge from hospital: a randomised controlled trial	Different comparators
Rutkowski S et al., 2023	Inpatient post-COVID-19 rehabilitation program featuring virtual reality-Preliminary results of randomized controlled trial	Different comparators
Karapolat H et al., 2007	Comparison of hospital-supervised exercise versus home-based exercise in patients after orthotopic heart transplantation: effects on functional capacity, quality of life, and psychological symptoms	Different comparators
Moore AD Jr et al. 2001	Maximal exercise as a countermeasure to orthostatic intolerance after spaceflight	Wrong outcome

Schneider S et al., 2013	The influence of exercise on prefrontal cortex activity and cognitive performance during a simulated space flight to Mars (MARS500)	Wrong outcome
Bernauer EM et al., 1994	Knee-joint proprioception during 30-day 6 degrees head-down bed rest with isotonic and isokinetic exercise training	Different comparators
Pires Peixoto R et al., 2020	Feasibility and safety of high-intensity interval training for the rehabilitation of geriatric inpatients (HIITERGY) a pilot randomized study	Different comparators
Moreno NA et al., 2019	Physiotherapist advice to older inpatients about the importance of staying physically active during hospitalisation reduces sedentary time, increases daily steps and preserves mobility: a randomised trial	Wrong outcome
Rao SJ et al., 2024	Phase 1 randomized trial of inpatient high-intensity interval training after major surgery	Wrong outcome
Sherrington C et al., 2003	A randomised trial of weight-bearing versus non-weight-bearing exercise for improving physical ability in inpatients after hip fracture	Different comparators
Lee SM et al., 1997	Upright exercise or supine lower body negative pressure exercise maintains exercise responses after bed rest	Wrong outcome
Scott JPR et al., 2020	Body size and its implications upon resource utilization during human space exploration missions	Wrong outcome
Mostert S et al., 2002	Effects of a short-term exercise training program on aerobic fitness, fatigue, health perception and activity level of subjects with multiple sclerosis	Wrong outcome
Peixoto TC et al., 2015	Early exercise-based rehabilitation improves health-related quality of life and functional capacity after acute myocardial infarction: a randomized controlled trial	Different comparators
Genc KO et al., 2010	Foot forces during exercise on the International Space Station	Incomplete endpoints
Guo N et al., 2018	Effect of Constraint Loading on the Lower Limb Muscle Forces in Weightless Treadmill Exercise	Wrong outcome
Rittweger J et al., 2007	Vertical jump performance after 90 days bed rest with and without flywheel resistive exercise, including a 180 days follow-up	Different comparators
Mudge AM et al., 2011	Exercise training in recently hospitalized heart failure patients enrolled in a disease management programme: design of the EJECTION-HF randomized controlled trial	Different comparators
Schoenrock B et al., 2024	Muscle stiffness indicating mission crew health in space	Wrong outcome
Katayama K et al., 2004	Acceleration with exercise during head-down bed rest preserves upright exercise responses	Wrong outcome
Cavanagh PR et al., 2010	Foot forces during typical days on the international space station	Wrong outcome
Brown CJ et al., 2006	Exercise program implementation proves not feasible during acute care hospitalization	Different comparators

Martínez-Velilla N et al., 2024	The impact of loneliness and social isolation on the benefits of an exercise program with hospitalised older adults	Different comparators
Behnke M et al., 2003	Clinical benefits of a combined hospital and home-based exercise programme over 18 months in patients with severe COPD	Participants under chronic treatment
Schneider SM et al., 2003	Training with the International Space Station interim resistive exercise device	Different comparators
Schneider S et al., 2010	Exercise as a countermeasure to psycho-physiological deconditioning during long-term confinement	Wrong outcome
Gade J et al., 2010	Protein-enriched, milk-based supplement to counteract sarcopenia in acutely ill geriatric patients offered resistance exercise training during and after hospitalisation: study protocol for a randomised, double-blind, multicentre trial	Different comparators
Brovold T et al., 2013	Older adults recently discharged from the hospital: effect of aerobic interval exercise on health-related quality of life, physical fitness, and physical activity	Different comparators
Lambrecht G et al., 2017	The role of physiotherapy in the European Space Agency strategy for preparation and reconditioning of astronauts before and after long duration space flight	Different comparators
Torres-Sánchez I et al., 2017	Effects of an Exercise Intervention in Frail Older Patients with Chronic Obstructive Pulmonary Disease Hospitalized due to an Exacerbation: A Randomized Controlled Trial	Participants under chronic treatment
Yekefallah L et al., 2019	Comparing the effects of upper limb and breathing exercises on six-minute walking distance among patients with chronic obstructive pulmonary disease: a three-group randomized controlled clinical trial	Participants under chronic treatment
Frett T et al., 2020	Motion sickness symptoms during jumping exercise on a short-arm centrifuge	Wrong outcome
Martinez Velilla N et al., 2023	Could a Tailored Exercise Intervention for Hospitalised Older Adults Have a Role in the Resolution of Delirium? Secondary Analysis of a Randomised Clinical Trial	Participants under chronic treatment
Pérez-Zepeda MU et al., 2022	The impact of an exercise intervention on frailty levels in hospitalised older adults: secondary analysis of a randomised controlled trial	Different comparators
Fregly BJ et al., 2015	Computational Prediction of Muscle Moments During ARED Squat Exercise on the International Space Station	Wrong outcome
Liu HY et al., 2022	Simulation study on the effect of resistance exercise on the hydrodynamic microenvironment of osteocytes in microgravity	Different comparators
Borges RC et al., 2014	Impact of resistance training in chronic obstructive pulmonary disease patients during periods of acute exacerbation	Participants under chronic treatment

Li X et al., 2015	Home-Based Exercise in Older Adults Recently Discharged From the Hospital for Cardiovascular Disease in China: Randomized Clinical Trial	Wrong outcome
Maggioni MA et al., 2012	Effects on body composition of different short-term rehabilitation programs in long-stay hospitalized elderly women	Different comparators
Owen PJ et al., 2020	Whey protein supplementation with vibration exercise ameliorates lumbar paraspinal muscle atrophy in prolonged bed rest	Different comparators
Ravizza M et al., 2023	MU-based classification of resistive exercises for real-time training monitoring on board the international space station with potential telemedicine spin-off	Different comparators
Claerbout M et al., 2012	Effects of 3 weeks' whole body vibration training on muscle strength and functional mobility in hospitalized persons with multiple sclerosis	Participants under chronic treatment
van Exter SH et al., 2025	Lessons learned from a combined, personalized lifestyle intervention in hospitalized patients at risk for sarcopenia: a feasibility study	Wrong outcome
Trappe S et al., 2007	Single muscle fiber function with concurrent exercise or nutrition countermeasures during 60 days of bed rest in women	Different comparators
Alkner BA et al., 2003	Effects of strength training, using a gravity-independent exercise system, performed during 110 days of simulated space station confinement.	Different comparators
Aydın T et al., 2014	Evaluation of the effectiveness of home based or hospital based calisthenic exercises in patients with multiple sclerosis	Participants under chronic treatment
McDowell K et al., 2017	Effectiveness of an exercise programme on physical function in patients discharged from hospital following critical illness: a randomised controlled trial (the REVIVE trial)	Wrong outcome
Yang Y et al., 2007	Hypergravity resistance exercise: the use of artificial gravity as potential countermeasure to microgravity	Different comparators
Belavý DL et al., 2011	Evidence for an additional effect of whole-body vibration above resistive exercise alone in preventing bone loss during prolonged bed rest	Different comparators
Maru S et al., 2019	One-year cost-effectiveness of supervised center-based exercise training in addition to a post-discharge disease management program for patients recently hospitalized with acute heart failure: The EJECTION-HF study	Different comparators
Santaularia N et al., 2013	Randomized clinical trial to evaluate the effect of a supervised exercise training program on readmissions in patients with myocardial ischemia: a study protocol	Wrong outcome
Selvadurai HC et al., 2002	Randomized controlled study of in-hospital exercise training programs in children with cystic fibrosis	Ineligible participant

Lee SM et al., 2005	Foot-ground reaction force during resistive exercise in parabolic flight	Different comparators
Katri Maria T et al., 2021	Effects of a home-based rehabilitation program in community-dwelling older people after discharge from hospital: A subgroup analysis of a randomized controlled trial	Different comparators
McGowan T et al., 2018	The effect of chair-based pedal exercises for older people admitted to an acute hospital compared to standard care: a feasibility study	Wrong outcome
Gade J et al., 2019	Protein supplementation combined with low-intensity resistance training in geriatric medical patients during and after hospitalisation: a randomised, double-blind, multicentre trial	Different comparators
Fitts RH et al., 2013	Effects of prolonged space flight on human skeletal muscle enzyme and substrate profiles	Wrong outcome
MacIntyre DL et al., 2005	Recovery of lower limb function following 6 weeks of non-weight bearing	Different comparators
Parker C et al., 2015	Randomized controlled trial of the effect of additional functional exercise during slow-stream rehabilitation in a regional center	Wrong outcome
Gojevic T et al., 2024	Effects of low vs. moderate intense resistance exercise training combined with endurance exercise training in patients with heart failure: a randomized clinical trial†	Different comparators
Tesch PA et al., 2005	Effects of 17-day spaceflight on knee extensor muscle function and size	Different comparators
Lau HM et al., 2005	A randomised controlled trial of the effectiveness of an exercise training program in patients recovering from severe acute respiratory syndrome	Different comparators
Kronborg L et al., 2017	Effectiveness of acute in-hospital physiotherapy with knee-extension strength training in reducing strength deficits in patients with a hip fracture: A randomised controlled trial	Wrong outcome
Said CM et al., 2012	Enhancing physical activity in older adults receiving hospital based rehabilitation: a phase II feasibility study	Wrong outcome
Dyson KS et al., 2005	WISE 2005: flow and nitroglycerin mediated dilation following 56 days of head down tilt bed rest with and without an exercise countermeasure	Different comparators
Beckmann M et al., 2021	Effect of an additional health-professional-led exercise programme on clinical health outcomes after hip fracture	Wrong outcome
Hirschhorn AD et al., 2012	Does the mode of exercise influence recovery of functional capacity in the early postoperative period after coronary artery bypass graft surgery? A randomized controlled trial	Different comparators
Qi R et al., 2023	Effects of perioperative exercise on cardiorespiratory endurance in children with congenital heart disease in plateau areas after surgical repair	Ineligible participant

Wade CE et al., 2004	Intensive exercise training suppresses testosterone during bed rest	Different comparators
Greenleaf JE et al., 1994	Isokinetic strength and endurance during 30-day 6 degrees head-down bed rest with isotonic and isokinetic exercise training	Wrong outcome
Caruso JF et al., 2008	Albuterol and exercise effects on ankle extensor strength during 40 days of unloading	Different comparators
Guinet P et al., 2009	WISE-2005: effect of aerobic and resistive exercises on orthostatic tolerance during 60 days bed rest in women	Wrong outcome
de Macedo RM et al., 2012	A periodized model for exercise improves the intra-hospital evolution of patients after myocardial revascularization: a pilot randomized controlled trial	Wrong outcome
Jentoft ES et al., 2020	Effect of information and exercise programmes after lumbar disc surgery: A randomized controlled trial	Different comparators
Stein R et al., 2009	Inspiratory muscle strength as a determinant of functional capacity early after coronary artery bypass graft surgery	Different comparators
Tamulevičiūtė-Prascienė et al., 2022	Effectiveness of additional resistance and balance training and telephone support program in exercise-based cardiac rehabilitation on quality of life and physical activity: Randomized control trial	Different comparators
Yoshimitsu K et al., 2010	Development of a training method for weightless environment using both electrical stimulation and voluntary muscle contraction	Different comparators
D'Andrea SE et al., 2005	Jumping in simulated and true microgravity: response to maximal efforts with three landing types	Different comparators
Watenpaugh DE et al., 2007	Lower body negative pressure exercise plus brief postexercise lower body negative pressure improve post-bed rest orthostatic tolerance	Different comparators
Aitken E et al., 2023	Exercise in adults admitted to hospital with diabetes-related foot ulcers: a pilot study of feasibility and safety	Participants under chronic treatment
Smolis-Bąk E et al., 2015	Hospital-based and telemonitoring guided home-based training programs: effects on exercise tolerance and quality of life in patients with heart failure (NYHA class III) and cardiac resynchronization therapy. A randomized, prospective observation	Different comparators
Namanja A et al., 2024	Delivering effective, comprehensive, multi-exercise component cardiac rehabilitation (CR) for chronic heart failure patients in low resource settings in sub-Saharan Africa: Queen Elizabeth Central Hospital-(QECH-CR) randomised CR study	Different comparators
Haines TP et al., 2004	Effectiveness of targeted falls prevention programme in subacute hospital setting: randomised controlled trial	Different comparators
Trombetti A et al., 2013	Effect of a multifactorial fall-and-fracture risk assessment and management program on gait and	Different comparators

	balance performances and disability in hospitalized older adults: a controlled study	
Hwang R et al., 2016	Timed Up and Go Test: A Reliable and Valid Test in Patients With Chronic Heart Failure	Wrong outcome
Friedl-Werner A et al., 2020	Exercise-induced changes in brain activity during memory encoding and retrieval after long-term bed rest	Wrong outcome
Iwasaki K et al., 2005	Hypergravity exercise against bed rest induced changes in cardiac autonomic control	Wrong outcome
Mulder ER et al., 2008	Characteristics of fast voluntary and electrically evoked isometric knee extensions during 56 days of bed rest with and without exercise countermeasure	Different comparators
Belin de Chantemele E et al., 2004	Does resistance exercise prevent body fluid changes after a 90-day bed rest?	Different comparators
Watenpaugh DE et al., 2000	Supine lower body negative pressure exercise during bed rest maintains upright exercise capacity	Wrong outcome
Dougherty CM et al., 2015	Prospective randomized trial of moderately strenuous aerobic exercise after an implantable cardioverter defibrillator	Wrong outcome
Treacy D et al., 2015	Additional standing balance circuit classes during inpatient rehabilitation improved balance outcomes: an assessor-blinded randomised controlled trial	Different comparators
Mentz RJ et al., 2013	Clinical characteristics, response to exercise training, and outcomes in patients with heart failure and chronic obstructive pulmonary disease: findings from Heart Failure and A Controlled Trial Investigating Outcomes of Exercise TraiNing (HF-ACTION)	Participants under chronic treatment
Salanova M et al., 2008	Molecular biomarkers monitoring human skeletal muscle fibres and microvasculature following long-term bed rest with and without countermeasures	Different comparators
Sato K et al., 2010	Aerobic exercise capacity and muscle volume after lower limb suspension with exercise countermeasure	Different comparators
Burtin C et al., 2009	Early exercise in critically ill patients enhances short-term functional recovery	Wrong outcome
Hodgson CL et al., 2016	A Binational Multicenter Pilot Feasibility Randomized Controlled Trial of Early Goal-Directed Mobilization in the ICU	Wrong outcome
Medrinal C et al., 2018	Comparison of exercise intensity during four early rehabilitation techniques in sedated and ventilated patients in ICU: a randomised cross-over trial	Different comparators
Lv X et al., 2024	Effect of perioperative rehabilitation exercise on postoperative outcomes in patients aged ≥65 years undergoing gastrointestinal surgery: A multicenter randomized controlled trial	Different comparators
Muthukrishnan R et al., 2021	Power walking based outpatient cardiac rehabilitation in patients with post-coronary angioplasty: Randomized control trial	Wrong outcome

Busch JC et al., 2012	Resistance and balance training improves functional capacity in very old participants attending cardiac rehabilitation after coronary bypass surgery	Different comparators
Skals S et al., 2018	Shoulder and arm muscle activity during elastic band exercises performed in a hospital bed	Different comparators
Germain P et al., 1995	Muscle strength during bedrest with and without muscle exercise as a countermeasure	Different comparators
Behnke M et al., 2000	Home-based exercise is capable of preserving hospital-based improvements in severe chronic obstructive pulmonary disease	Participants under chronic treatment
Kuzmik A et al., 2021	Physical Activity in Hospitalized Persons With Dementia: Feasibility and Validity of the MotionWatch 8	Participants under chronic treatment
de Azevedo JRA et al., 2021	High-protein intake and early exercise in adult intensive care patients: a prospective, randomized controlled trial to evaluate the impact on functional outcomes	Different comparators
Campo G et al., 2020	Exercise intervention improves quality of life in older adults after myocardial infarction: randomised clinical trial	Wrong outcome
Eggmann S et al., 2018	Effects of early, combined endurance and resistance training in mechanically ventilated, critically ill patients: A randomised controlled trial	Different comparators
Connolly B et al., 2015	Exercise-based rehabilitation after hospital discharge for survivors of critical illness with intensive care unit-acquired weakness: A pilot feasibility trial	Wrong outcome
Vallier JM et al., 2023	Randomized controlled trial of home-based vs. hospital-based pulmonary rehabilitation in post COVID-19 patients	Different comparators
Wu WX et al., 2020	Effect of Early and Intensive Rehabilitation after Ischemic Stroke on Functional Recovery of the Lower Limbs: A Pilot, Randomized Trial	Different comparators
Hirschhorn AD et al., 2008	Supervised moderate intensity exercise improves distance walked at hospital discharge following coronary artery bypass graft surgery--a randomised controlled trial	Wrong outcome
Sherrington C et al., 2009	Minimising disability and falls in older people through a post-hospital exercise program: a protocol for a randomised controlled trial and economic evaluation	Participants under chronic treatment
Timonen L et al., 2006	Effects of a group-based exercise program on functional abilities in frail older women after hospital discharge	Wrong outcome
Widrick JJ et al., 1999	Effect of a 17 day spaceflight on contractile properties of human soleus muscle fibres	Different comparators
Haines TP et al., 2009	Effectiveness of a video-based exercise programme to reduce falls and improve health-related quality of life among older adults discharged from hospital: a pilot randomized controlled trial	Different comparators

O'Neill B et al., 2014	Effectiveness of a programme of exercise on physical function in survivors of critical illness following discharge from the ICU: study protocol for a randomised controlled trial (REVIVE)	Wrong outcome
Foley A et al., 2011	Effectiveness of once-weekly gym-based exercise programmes for older adults post discharge from day rehabilitation: a randomised controlled trial	Different comparators
Reeves ND et al., 2005	Influence of 90-day simulated microgravity on human tendon mechanical properties and the effect of resistive countermeasures	Wrong outcome
Fransen M et al., 2017	Post-Acute Rehabilitation After Total Knee Replacement: A Multicenter Randomized Clinical Trial Comparing Long-Term Outcomes	Wrong outcome
Vogler CM et al., 2012	Evidence of detraining after 12-week home-based exercise programs designed to reduce fall-risk factors in older people recently discharged from hospital	Different comparators
Vogler CM et al., 2009	Reducing risk of falling in older people discharged from hospital: a randomized controlled trial comparing seated exercises, weight-bearing exercises, and social visits	Different comparators
Delgado B et al., 2022	The effects of early rehabilitation on functional exercise tolerance in decompensated heart failure patients: Results of a multicenter randomized controlled trial (ERIC-HF study)	Different comparators
Mudge AM et al., 2008	Exercising body and mind: an integrated approach to functional independence in hospitalized older people	Wrong outcome
Stewart S et al., 1998	Effects of a home-based intervention among patients with congestive heart failure discharged from acute hospital care	Wrong outcome
Nickels MR et al., 2017	Critical Care Cycling Study (CYCLIST) trial protocol: a randomised controlled trial of usual care plus additional in-bed cycling sessions versus usual care in the critically ill	Different comparators
Graves N et al., 2009	Cost-effectiveness of an intervention to reduce emergency re-admissions to hospital among older patients	Different comparators
