# Supplementary material for: Disuse‐Induced Muscle Atrophy and Muscle Weakness From Hospitalization to Spaceflight: Exercise Succeeds in Prevention and Treatment—A Meta‐Analysis
Source: J Cachexia Sarcopenia Muscle. 2026 Apr 15;17(2):e70259. doi: 10.1002/jcsm.70259 (PMC13080877; doi:10.1002/jcsm.70259)
Supplement: Supplementary file 9 — Data S4: Supporting information. [file JCSM-17-e70259-s002.pdf]

Cochrane Risk of Bias Assessment for Randomised Controlled Trials and Non-Randomised Controlled Trials (low, high, unclear).

Risk of Bias Assessment for Randomised Controlled Trials						
**Study**	**Random sequence generation**	**Allocation concealment**	**Blinding of participants and personnel**	**Blinding of outcome assessment**	**Incomplete outcome data addressed**	**Selective reporting**
Akima et al. 2000	**Low risk**	**High risk**	**Unclear risk**	**Unclear risk**	**Low risk**	**Low risk**
Alkner and Tesch 2004	**Low risk**	**Low risk**	**Unclear risk**	**Low risk**	**Low risk**	**Low risk**
Asteasu et al. 2019	**Low risk**	**Low risk**	**Low risk**	**Unclear risk**	**Low risk**	**Low risk**
Asteasu et al. 2020	**Low risk**	**Low risk**	**Low risk**	**Unclear risk**	**Low risk**	**Low risk**
Bamman et al. 1998	**Low risk**	**Low risk**	**Unclear risk**	**Low risk**	**Low risk**	**Low risk**
Belavý et al. 2010	**Low risk**	**Low risk**	**Unclear risk**	**Low risk**	**Low risk**	**Low risk**
Belavý et al. 2016	**Low risk**	**Low risk**	**Unclear risk**	**Low risk**	**Low risk**	**Low risk**
Cadore et al. 2023	**Low risk**	**Low risk**	**Low risk**	**Unclear risk**	**Low risk**	**Low risk**
Echeverria et al. 2020	**Low risk**	**Low risk**	**Unclear risk**	**High risk**	**Low risk**	**Low risk**
Hacker et al. 2017	**Low risk**	**Low risk**	**Low risk**	**Unclear**	**Low risk**	**Low risk**
Holt et al. 2016	**Low risk**	**Low risk**	**Unclear risk**	**Low risk**	**Low risk**	**Low risk**
Martínez-Velilla et al. 2018	**Low risk**	**Low risk**	**Low risk**	**Unclear risk**	**Low risk**	**Low risk**
Morris et al. 2016	**Low risk**	**Low risk**	**Unclear risk**	**Unclear risk**	**Low risk**	**Low risk**
Mulder et al. 2015	**Low risk**	**High risk**	**Unclear risk**	**Unclear risk**	**Low risk**	**Low risk**
Ogawa et al. 2020	**Low risk**	**Low risk**	**Low risk**	**Unclear risk**	**Low risk**	**Low risk**
Ploutz-Snyder et al. 2018	**Low risk**	**High risk**	**Low risk**	**Low risk**	**Low risk**	**Low risk**
Schneider et al. 2016	**Low risk**	**Unclear risk**	**Low risk**	**Unclear**	**Low risk**	**Low risk**
Rittweger et al. 2007	**Low risk**	**Low risk**	**Unclear risk**	**Low risk**	**Low risk**	**Low risk**

Robin et al. 2022	**Low risk**	**Low risk**	**Unclear risk**	**Low risk**	**Low risk**	**Low risk**
Timonen et al. 2002	**Low risk**	**Low risk**	**Low risk**	**Low risk**	**Low risk**	**Low risk**
Trappe et al. 2004	**Low risk**	**Low risk**	**Unclear risk**	**Unclear risk**	**Low risk**	**Low risk**
Non-Randomised Controlled Trials: ROBIN-1						
**Study**	**Bias in selection of participants into the study**	**Bias in classification of interventions**	**Bias due to deviations from intended interventions**	**Bias due to missing data**	**Bias in measurement of outcomes**	**Overall bias**
English et al. 2020	**Low risk**	**Moderate risk**	**Critical risk**	**Low risk**	**Low risk**	**Low risk**
Loehr et al. 2011	**Low risk**	**Moderate risk**	**Low risk**	**Low risk**	**Low risk**	**Low risk**
Mulavara et al. 2018	**Low risk**	**Low risk**	**Low risk**	**Low risk**	**Low risk**	**Low risk**
Petersen et al. 2016	**Low risk**	**Moderate risk**	**Moderate risk**	**Low risk**	**Low risk**	**Low risk**
Scott et al. 2023	**Low risk**	**Low risk**	**Low risk**	**Low risk**	**Low risk**	**Low risk**
Sibonga et al. 2019	**Low risk**	**Low risk**	**Low risk**	**Low risk**	**Low risk**	**Low risk**
Smith et al. 2012	**Low risk**	**Critical risk**	**Critical risk**	**Low risk**	**Low risk**	**Low risk**
Trappe et al. 2009	**Low risk**	**Critical risk**	**Low risk**	**Low risk**	**Low risk**	**Low risk**
