# Supplementary figures and images for: Disuse‐Induced Muscle Atrophy and Muscle Weakness From Hospitalization to Spaceflight: Exercise Succeeds in Prevention and Treatment—A Meta‐Analysis
Source: J Cachexia Sarcopenia Muscle. 2026 Apr 15;17(2):e70259. doi: 10.1002/jcsm.70259 (PMC13080877; doi:10.1002/jcsm.70259)

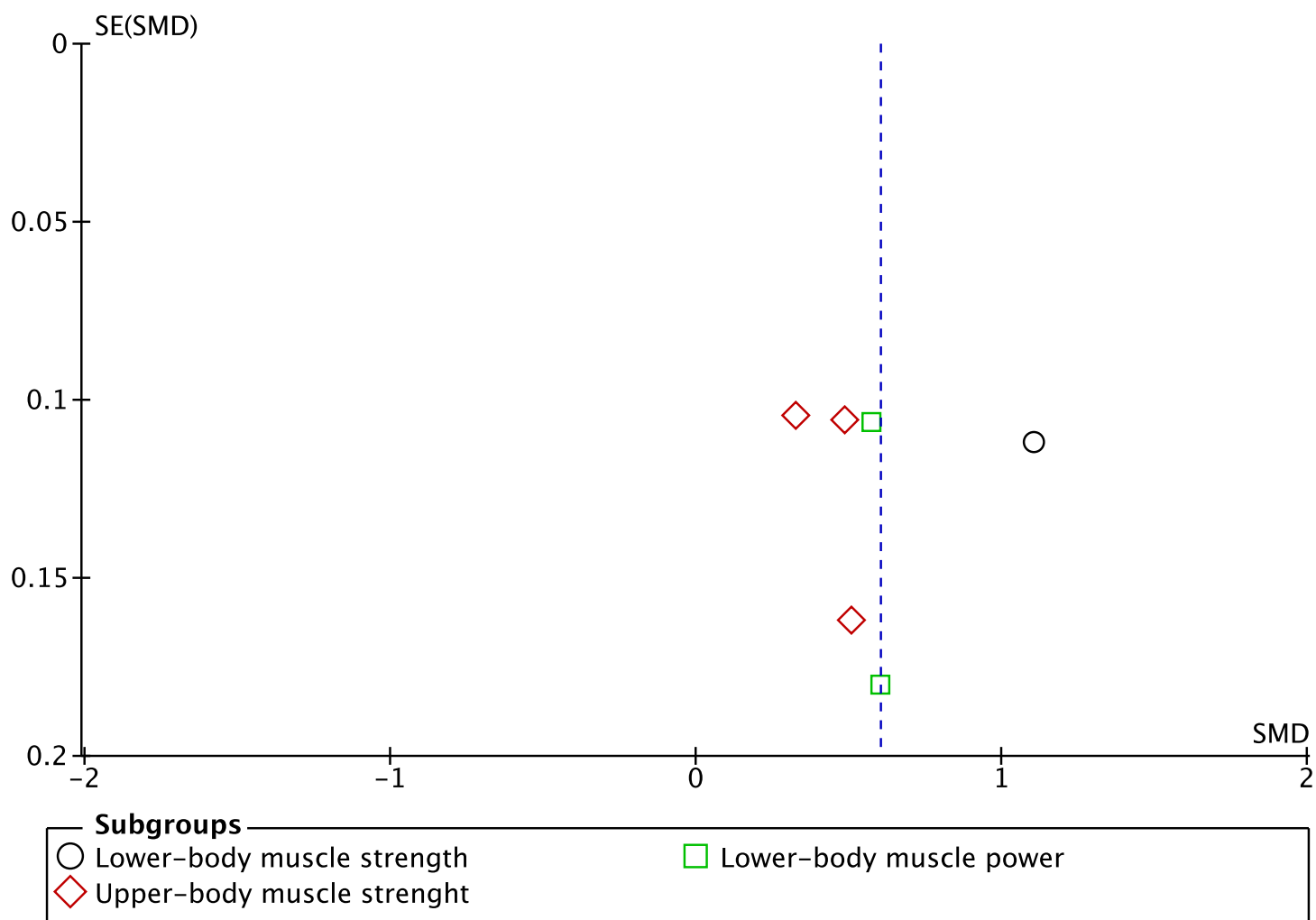

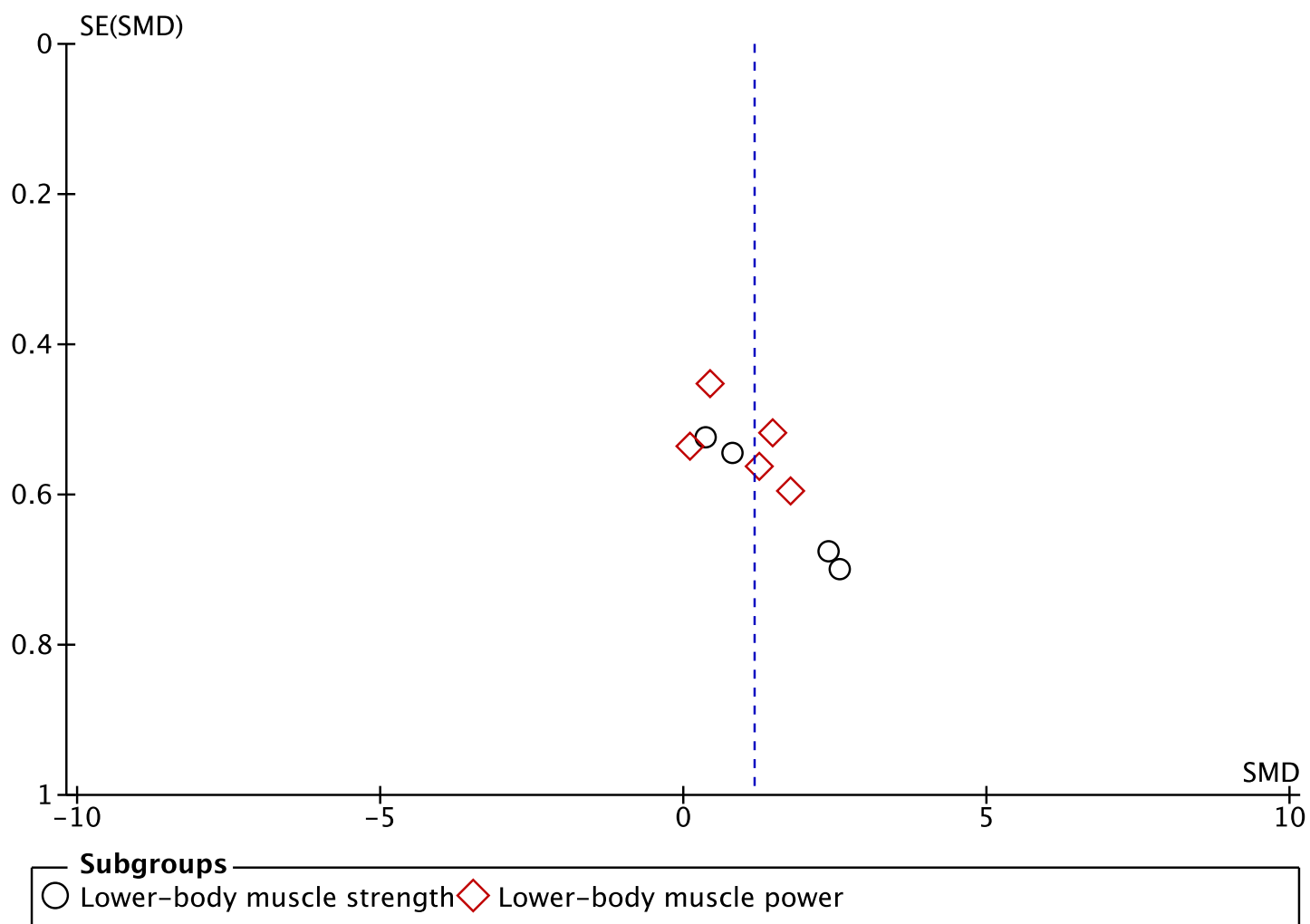

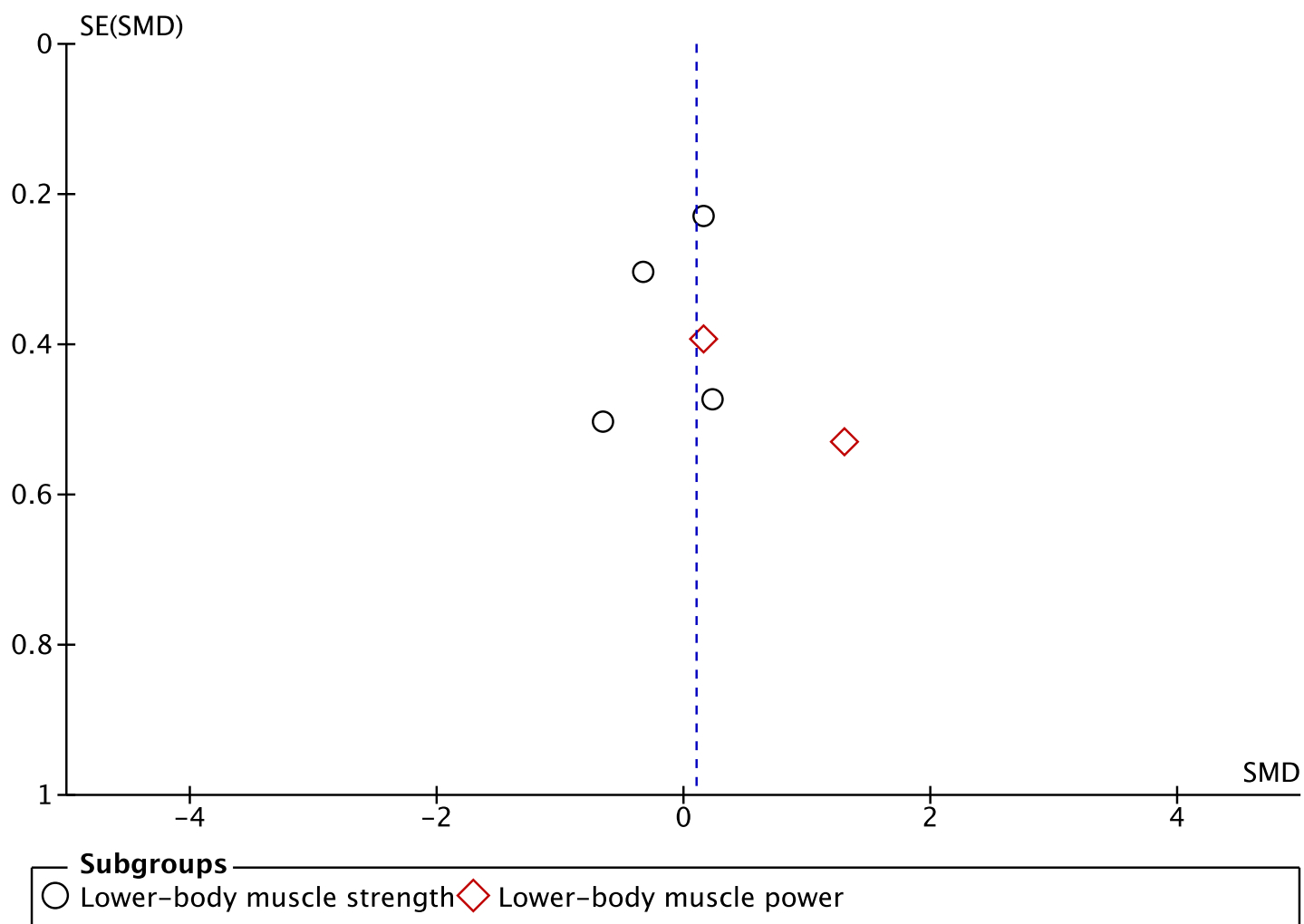

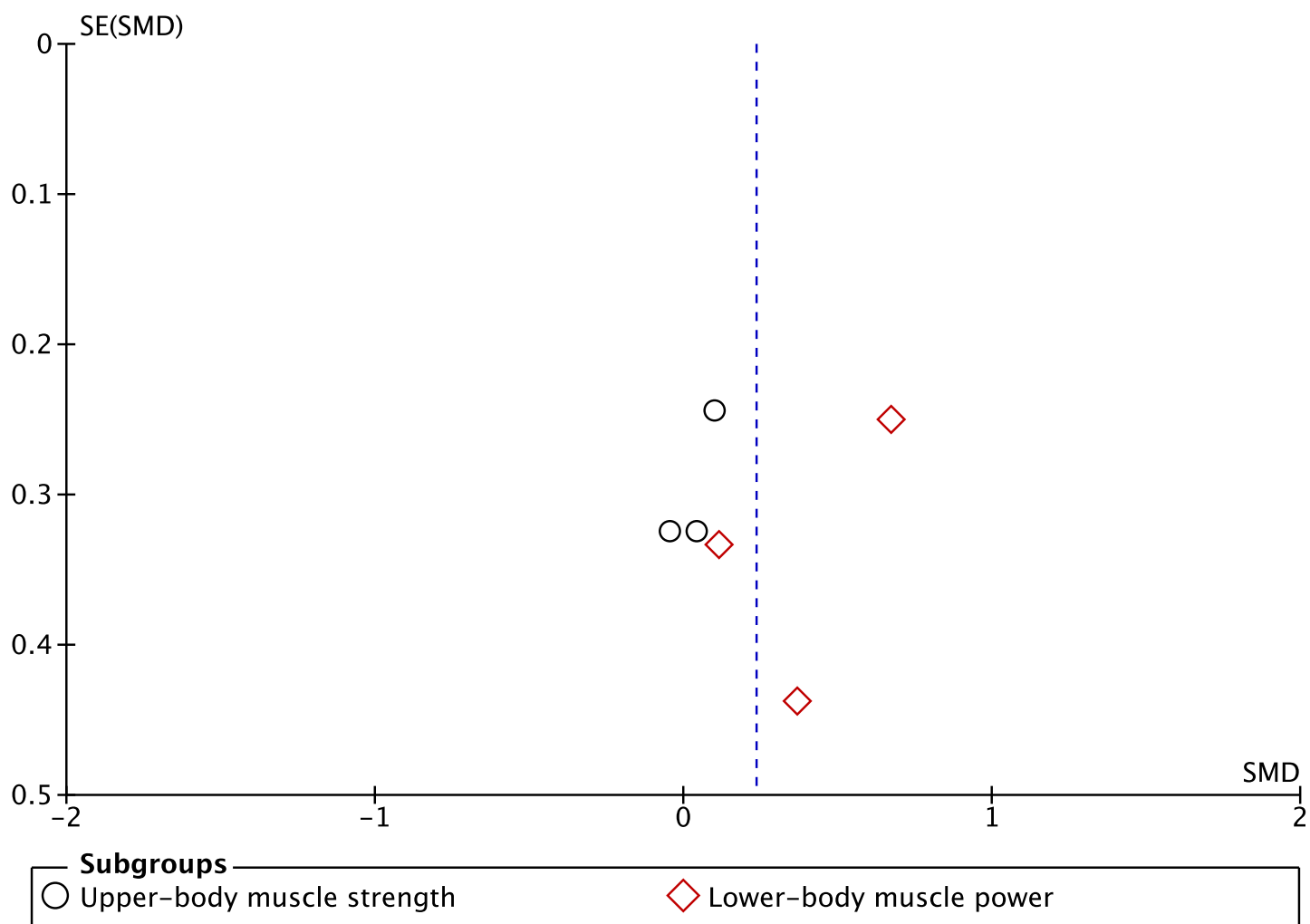

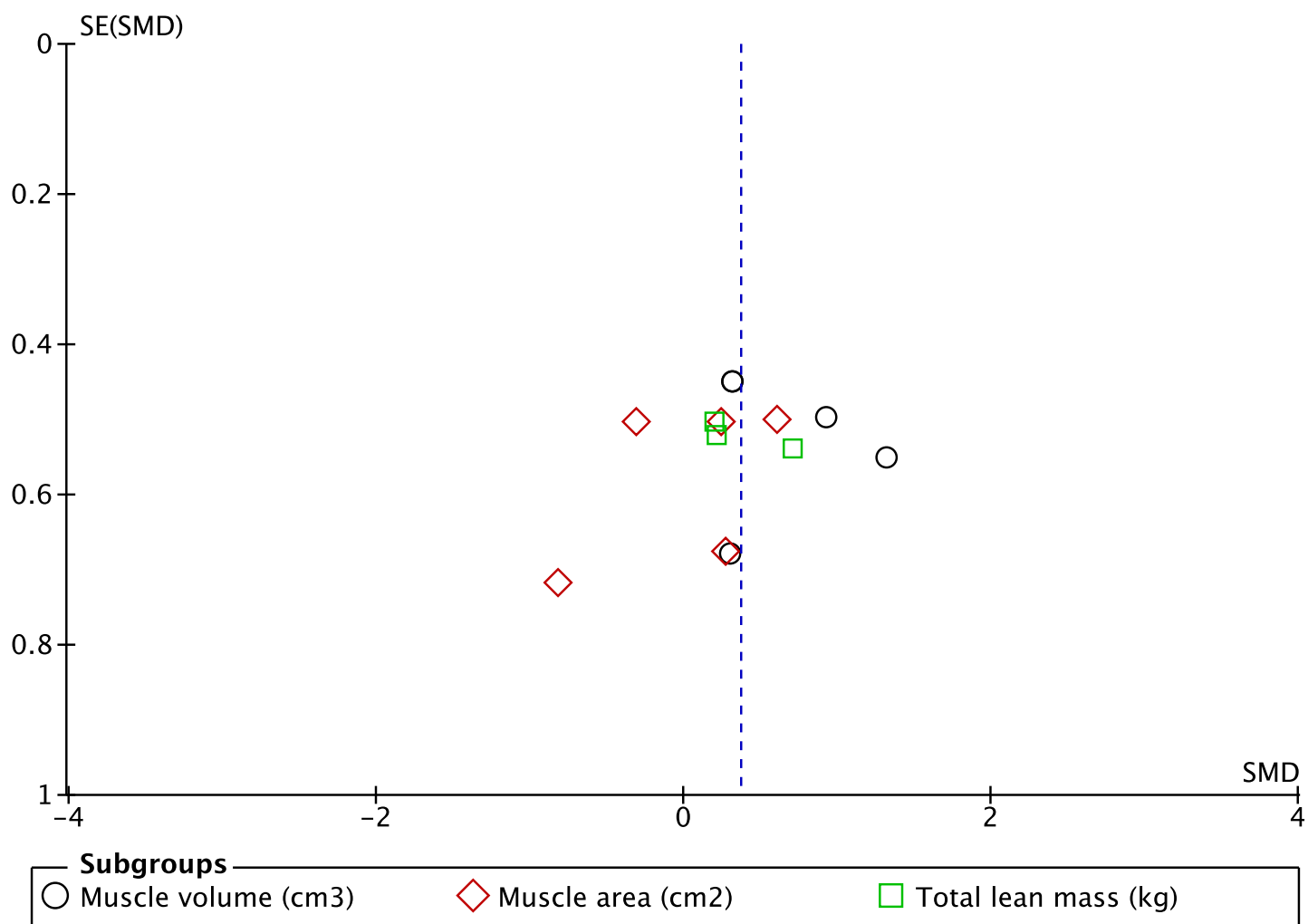

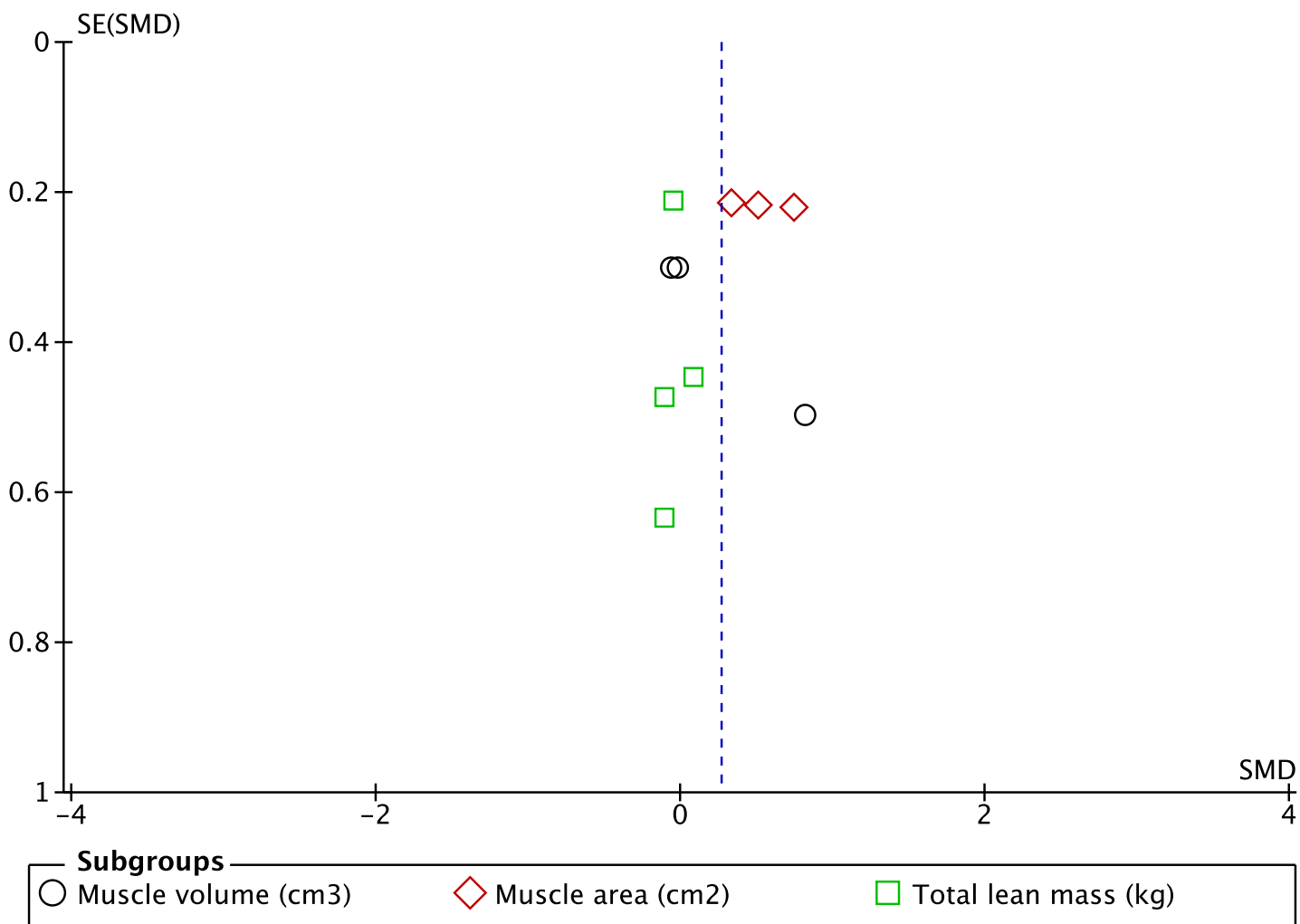

Supplement: Supplementary file 10 — Data S5: Supporting information. [file JCSM-17-e70259-s006.pdf]
