# Supplementary material for: Disuse‐Induced Muscle Atrophy and Muscle Weakness From Hospitalization to Spaceflight: Exercise Succeeds in Prevention and Treatment—A Meta‐Analysis
Source: J Cachexia Sarcopenia Muscle. 2026 Apr 15;17(2):e70259. doi: 10.1002/jcsm.70259 (PMC13080877; doi:10.1002/jcsm.70259)
Supplement: Supplementary file 11 — Data S6: Supporting information. [file JCSM-17-e70259-s003.pdf]

Supplemental Material 6. Moderators of muscle function and mass

To explore potential sources of between-study heterogeneity, random-effects meta-regression analyses were conducted using intervention duration (days) and mean participant age (years) as continuous moderators. Meta-regressions were performed separately for muscle strength, muscle power, and muscle mass outcomes. Effect sizes were expressed as Hedges' g and weighted by their corresponding within-study standard errors, using restricted maximum likelihood (REML) estimation with Knapp–Hartung adjustment. For each model, were reported the regression coefficient (β), 95% confidence interval (95% CI), p-value, and measures of residual heterogeneity (τ^2 and I^2). The number of studies contributing to each meta-regression (k) is reported to reflect available statistical power. Meta-regression analyses were conducted in StataSE version 14.

Moderators of muscle strength and muscle mass in disuse-induced atrophy.

Moderators	β	95% CI	p-value	τ^2 residual	I^2 residual	k
Muscle strength						
Age (years)	-0.0001	-0.0078 to 0.0075	0.974	0.127	57.55	30
Duration of intervention (days)	-0.0018	-0.0046 to 0.0009	0.181	0.078	55.35	30
Muscle power						
Age (years)	-0.0034	-0.0234 to 0.0165	0.710	0.427	68.47	13
Duration of intervention (days)	0.0013	-0.0084 to 0.0111	0.769	0.429	69.44	13
Muscle mass						
Age (years)	-0.0301	-0.0753 to 0.0149	0.173	0	0	16
Duration of intervention (days)	-0.0026	-0.0061 to 0.0009	0.135	0	0	16

Note: k = number of studies included for each moderator.
